# Sleep is required to consolidate odor memory and remodel olfactory synapses

**DOI:** 10.1016/j.cell.2023.05.006

**Published:** 2023-06-02

**Authors:** Rashmi Chandra, Fatima Farah, Fernando Muñoz-Lobato, Anirudh Bokka, Kelli L. Benedetti, Chantal Brueggemann, Mashel Fatema A. Saifuddin, Julia M. Miller, Joy Li, Eric Chang, Aruna Varshney, Vanessa Jimenez, Anjana Baradwaj, Cibelle Nassif, Sara Alladin, Kristine Andersen, Angel J. Garcia, Veronica Bi, Sarah K. Nordquist, Raymond L. Dunn, Vanessa Garcia, Kateryna Tokalenko, Emily Soohoo, Fabiola Briseno, Sukhdeep Kaur, Malcolm Harris, Hazel Guillen, Decklin Byrd, Brandon Fung, Andrew E. Bykov, Emma Odisho, Bryan Tsujimoto, Alan Tran, Alex Duong, Kevin C. Daigle, Rebekka Paisner, Carlos E. Zuazo, Christine Lin, Aarati Asundi, Matthew A. Churgin, Christopher Fang-Yen, Martina Bremer, Saul Kato, Miri K. VanHoven, Noëlle D. L’Étoile

**Affiliations:** 1 Department of Cell and Tissue Biology, University of California, San Francisco, San Francisco, CA 94143, USA; 2 Department of Biological Sciences, San José State University, San José, CA 95192, USA; 3 Department of Neurology, University of California, San Francisco, San Francisco, CA 94158, USA; 4 Department of Bioengineering, School of Engineering and Applied Science, University of Pennsylvania, Philadelphia, PA 19104, USA; 5 Department of Neuroscience, Perelman School of Medicine at the University of Pennsylvania, Philadelphia, PA 19104, USA; 6 Department of Mathematics and Statistics, San José State University, San José, CA 95192, USA; 7 These authors contributed equally; 8 These authors contributed equally; 9 Lead contact

## Abstract

Animals with complex nervous systems demand sleep for memory consolidation and synaptic remodeling. Here, we show that, although the *Caenorhabditis elegans* nervous system has a limited number of neurons, sleep is necessary for both processes. In addition, it is unclear if, in any system, sleep collaborates with experience to alter synapses between specific neurons and whether this ultimately affects behavior. *C. elegans* neurons have defined connections and well-described contributions to behavior. We show that spaced odor-training and post-training sleep induce long-term memory. Memory consolidation, but not acquisition, requires a pair of interneurons, the AIYs, which play a role in odor-seeking behavior. In worms that consolidate memory, both sleep and odor conditioning are required to diminish inhibitory synaptic connections between the AWC chemosensory neurons and the AIYs. Thus, we demonstrate in a living organism that sleep is required for events immediately after training that drive memory consolidation and alter synaptic structures.

## In brief

A sleep-like state is required for memory consolidation and synaptic remodeling, even in organisms with relatively small nervous systems, such as *C. elegans*, in which it is possible to examine how sleep affects individual neurons and their connections.

## Graphical Abstract

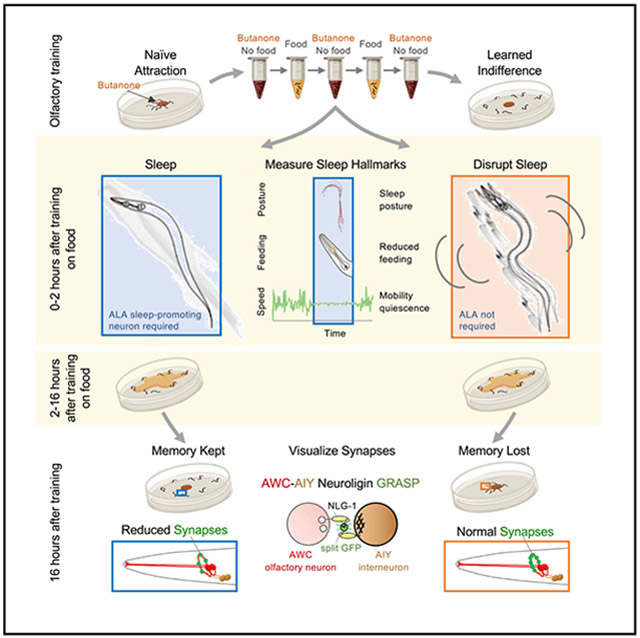

## INTRODUCTION

Understanding how the global brain state in sleep promotes consolidation of a specific memory within dedicated circuits is one of the foremost challenges in biology.^1^ A system with a simple, well-defined circuit that depends on sleep for consolidation would aid in addressing this question with cellular and synaptic resolution. Animals ranging from humans to flies to sea hares have been shown to require sleep to consolidate memory.^2-9^ These organisms have large, complex brains, ranging from approximately 200,000 neurons in *Drosophila*^10^ to eighty-six billion in humans.^11-14^ *C. elegans* has a compact nervous system with only 302 neurons.^15^ The nervous system is also well characterized; the functions of many individual neurons have been identified and the entire synaptic connectome has been elucidated.^16-18^ Thus, *C. elegans* provides a unique opportunity to examine how sleep might affect connections between individual neurons that control behaviors altered by learning and memory.

The hallmarks of sleep are conserved in *C. elegans*: periods of quickly reversed immobility, increased arousal threshold, homeostatic compensation, stereotypical posture, and broadly altered patterns of neuronal activity.^19-22^ The *C. elegans* sleep-promoting neurons ALA, which is required for stress-induced sleep, and RIS, which is required for developmentally timed sleep,^23^ release conserved neuropeptides that engage GABAergic pro-sleep circuits^24-26^ and downstream pathways.^21,27,28^ As observed in other animals, this global brain state in *C. elegans* shows reduced neuronal activity.^19,20^ We can thus address how this state affects memory by examining the cells and specific connections that hold a memory.

Memory is consolidated at the synaptic, cellular, and systems levels; however, the role of sleep at any of these levels is unclear.^29-31^ Learning and memory are thought to transition from activity-induced changes in transcription and synaptic signaling^32^ into physical changes in synaptic structures within circuits affected by training, a process called synaptic consolidation.^33^ The transcriptional changes that drive long term, consolidated memory require cAMP response element binding protein (CREB).^34,35^ Work in mammalian systems and *Drosophila* established the evolutionary conservation of these mechanisms.^2,33,36^ Systems consolidation occurs when the physical manifestation of the memory is moved to a different brain region where it is stored, and its conservation has also been demonstrated.^30,37^ During sleep, synapses can be downscaled across broad regions of the brain in many systems, a process posited to maintain synaptic strength within a functional range.^38,39^ Despite these important findings, an understanding of sleep’s function in consolidating long-term memory at the cellular and synaptic levels remains elusive.

In this study, we first asked whether *C. elegans*, similar to organisms with more complex nervous systems, requires sleep for long-term memory consolidation. Previous studies have elucidated the identities of neurons required for butanone chemosensation^40-43^ and olfactory learning.^16,44-46^ Butanone is sensed by AWC^ON^, one of the two AWC olfactory neurons,^47^ which primarily form synapses with three pairs of interneurons: the AIYs, AIAs, and AIBs.^15,18,40,41,43^ Using a training paradigm adapted from Kauffman and colleagues,^48^ we show that olfactory memory is dependent upon post-training ALA-dependent sleep; if sleep is disrupted during the critical period by either mechanical disturbance or removal from food, the animals do not consolidate memory.

We next show that odor training and post-training sleep modify the butanone olfactory circuit. Either AIB or AIY interneurons are sufficient for butanone learning. However, after sleep, AIYs are more important for memory consolidation. We used the transsynaptic marker neuroligin-1 (NLG-1) GFP reconstitution across synaptic partners (GRASP)^49^ to visualize synapses between AWC chemosensory neurons and the AIY interneurons. We find that butanone-trained animals have significantly reduced AWC-AIY connections when compared with their control buffer-trained counterparts 16 h after training. Interrupting sleep immediately after training abolishes this synaptic reduction. During the first 2 h after training, synapses in both control buffer- and butanone-trained animals are reduced, but during the following 14 h, AWC-AIY synaptic levels in butanone- and buffer-trained animals become distinct. Our study reveals that sleep is required for odor memory consolidation and sleep-dependent synaptic structural plasticity in one of the simplest nervous systems. This work also provides a new level of precision and granularity in understanding how specific cells required for memory and their connections are modulated by odor training and sleep.

## RESULTS

### Olfactory conditioning induces sleep and long-lasting memory

**Figure 1.**
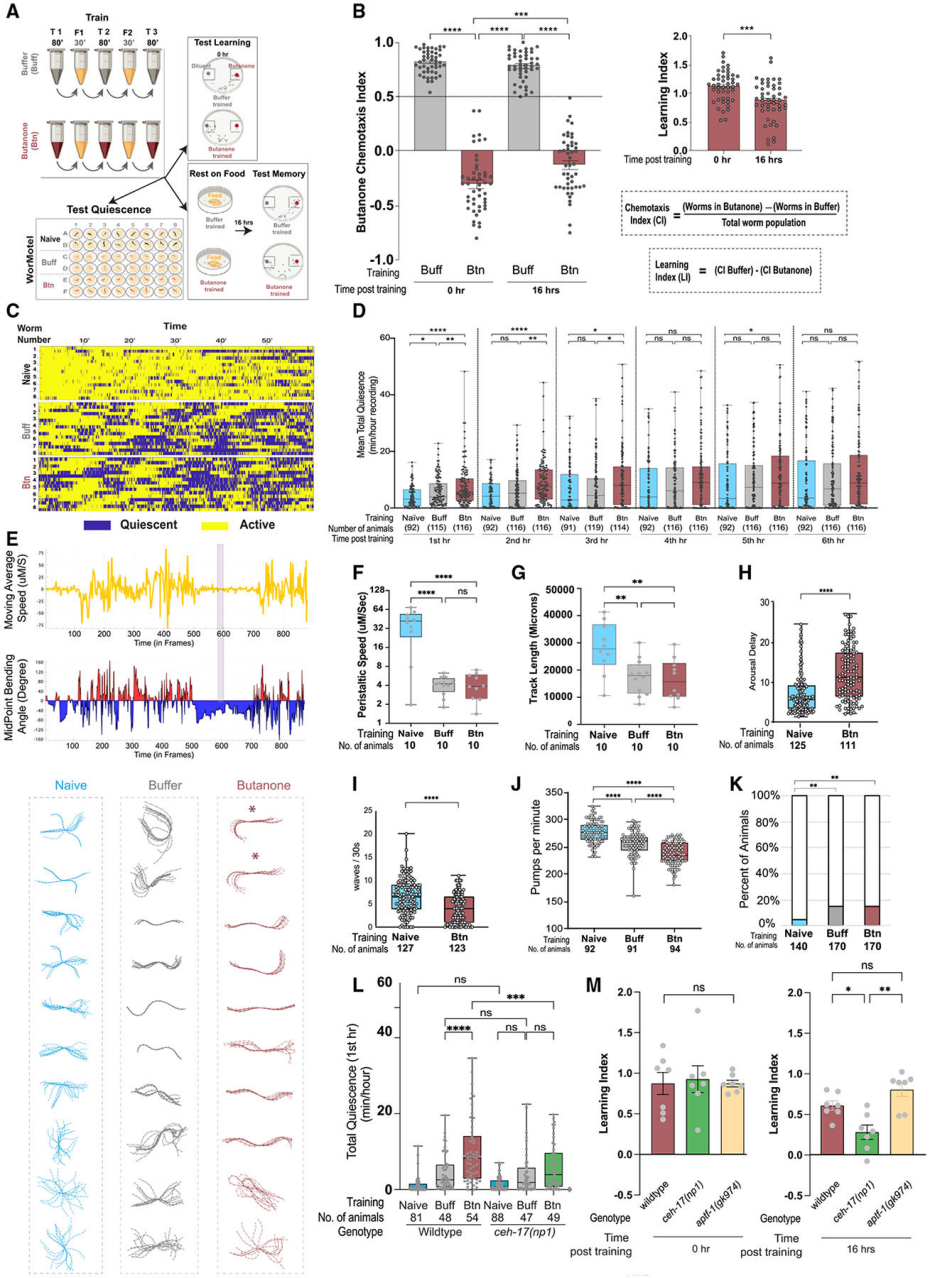
Spaced training paradigm induces long-lasting memory and quiescence that exhibits hallmarks of sleep (A) Training and subsequent analysis. Wild-type populations were subjected to repeated, spaced training with either butanone or buffer (control), then split into thirds and tested for learning (chemotaxis assays), placed on plates with food (*E. coli*) for 16 h, then tested for memory (chemotaxis assays) or single animals were loaded in individual wells of a WorMotel device containing food immediately after training. (B) Learning and 16-h memory. Chemotaxis indices (CIs) and learning indices (LIs) were calculated as indicated. CI and LI data points represent a trial of >50 animals on a separate day. n = 47 trials. One-way ANOVA was performed on the CIs and two-tailed t test on the LIs (***p < 0.001, ****p < 0.0001). (C) Quiescence analysis. Movement was imaged at 1 frame per 3 s. Raster plot showing activity (yellow) and quiescence (blue, >10 s movement) of naive, buffer- or butanone-trained individuals at the indicated time. Each row represents one individual animal. (D) Quiescence over 6 h. Each data point represents an animal’s mean total quiescence (minutes) over the hour indicated, and numbers of animals are indicated below. ****p < 0.0001, ***p < 0.001, **p < 0.01, *p < 0.05, one-way ANOVA with Bonferroni’s correction, n = 7 trials. (E) Posture analysis. Top, (yellow trace) example of one animal’s moving average speed and below, blue and red histogram of midpoint-bending angle in the hour after training. Shaded bar indicates a 10-frame period with the lowest speed and least bending angle. Bottom, 10 consecutive frames from this period were captured, animals were skeletonized, and their skeletons aligned to their midpoints and overlayed. (F) Movement speed. The speed of each animal in (E) is plotted. (G) Track length. The distance (millimeter) each animal in (E) traveled is plotted. (F and G) ****p < 0.0001, one-way ANOVA with Bonferroni’s multiple correction. (H) Arousal delay. Time (seconds) before blue LED light flashes and 1.2 kHz of vibrations evoked a complete sinusoidal escape wave. Data show for individual animals in three separate trials. (I) Activity following arousal. The number of sinusoidal waves completed in 30 s after exposure to blue LED (light emitting diode) light and 1.2 kHz vibrations stimuli. Additional animals from videos in (H) were analyzed. (H and I) ****p < 0.0001, ***p < 0.001, Mann-Whitney U test. (J) Feeding rate. Each point indicates the pharyngeal pumps per minute for one animal, 5 trials. One-way ANOVA with Bonferroni’s multiple correction, ****p < 0.0001. (K) Feeding quiescence. Fraction of immobile animals on food not pumping for at least 4 s (colored bars) within a minute of observation. Z test with Hochberg correction, **p < 0.01. Error is standard error of the proportiosn. (L) ALA-defective ceh-17 mutants are less quiescent than wild types after butanone training. Mean total quiescence in the first hour after training for naive, buffer- and butanone-trained wild-type and ALA-defective ceh-17(np1) animals examined in the WorMotel. n = 5 trials. (M) ALA-defective ceh-17 mutants consolidate less memory than wild types or RIS-defective mutants. LIs of wild-type, *ceh-17(np1)* and *aptf-1(gk974)* mutant strains 0 and 16 h after training. n = 7 trials. (L and M) ****p < 0.0001, ***p < 0.001, **p < 0.01, *p < 0.05 and (ns) is p > 0.05, one-way ANOVA of LIs, followed by Bonferroni’s multiple correction. All error bars are ±SEM unless mentioned. Figures S1, S2, and S3, Table S1 and S2, and Video S1 are related to Figure 1.

To understand how long-lasting memories are formed and retained, we adapted a spaced, repeated conditioning paradigm^50^ (Figure 1A) in which *C. elegans* learn to ignore butanone, an innately attractive odor emitted by nutritious^51^ and pathogenic bacteria.^52^ In this paradigm, the negative unconditioned stimulus is removal from food and is paired with either dilute butanone or control buffer. Attraction is quantified using a chemotaxis assay that provides a chemotaxis index (CI, Figures 1A and 1B).^53^ Learning is defined as the difference in a population’s attraction to butanone after training with butanone as compared with buffer-training (see inset in Figure 1B). We then asked how long this memory lasts. We found that the memory was kept 16 h after training (Figure 1B), as previously observed.^50^ In fact, we found that this memory persists up to 24 h (Figures S1A and S1B).

Butanone conditioning did not interfere with detection of other odors; it did not affect chemotaxis to the food-associated odors diacetyl (S1C) or benzaldehyde (Figures S1D and S1E). Diacetyl is sensed by the AWA neurons and benzaldehyde by both AWCs.^54^ Thus, memory is specific for the training odor, butanone.

### Animals are movement quiescent after training

After training, the animals appeared quiescent; therefore, we asked whether *C. elegans* sleep after conditioning by assessing conserved sleep features: decreased movement, stereotypical posture, reduced feeding, and increased arousal latency.^21^ We first assessed individual animals’ movement using the WorMotel, a video-based tool^55^ (Figure 1A; Video S1). Individual animals are placed into the bacteria-filled WorMotel wells (Video S1), and the automated system measures pixel displacement to quantify 10-s bouts with no movement.^55^ By summing the duration of these quiescent bouts each hour for 6 h, we determined that trained animals show significantly more quiescence than naive during the first hour after training. This difference subsequently decreases, as naive and buffer-trained animals become more quiescent (Figures 1C and 1D).

### Trained animals exhibit stereotypical postures during quiescent bouts

We then asked if trained animals showed a stereotypical posture during quiescence. Images of naive, buffer- and butanone-trained animals undergoing quiescent bouts were captured every 10 s, skeletonized and aligned to the animals’ midpoints using WormLab analysis software. Two of the ten butanone-trained animals were largely straight with a curve in the anterior region, resembling the canonical “shepherd’s crook” sleep posture^22,56,57^ (Figure 1E). We observed a similar percentage (Figure S2C) in animals induced to sleep using heat shock.^58^ By contrast, none of the buffer-trained or naive animals showed this posture (Figure 1E). Consistent with their increased quiescence, buffer- and butanone-trained animals exhibited less backward and forward movement, termed peristaltic movement (Figure 1F), and shorter track lengths (Figure 1G) than naive animals.

### Animals take longer to arouse after training

Next, we asked if trained animals exhibited increased arousal latency,^59^ by stimulating animals with a blue light pulse (a noxious stimulus) in conjunction with mechanical vibrations (1 kHz frequency) from a piezoelectric buzzer and quantifying the time until each animal executed a sinusoidal escape movement. Butanone-trained animals took longer to arouse than naive (Figure 1H), and had a similar latency to that described during sleep.^59^ Trained animals also executed fewer body bends after the stimulus was removed (Figure 1I), which may reflect a sleep pressure incurred by the stimulation.

### Animals feed less after training

We next examined feeding in animals after training. The rate of pharyngeal pumping was significantly decreased in buffer- and butanone-trained populations when compared with naive (Figure 1J). We also assessed the proportion of still animals that paused pumping^58^ and found that 15% of butanone- and buffer-trained animals paused, which is significantly more than we observed in naive animals (Figure 1K), but less than we observed in heat-shocked animals (Figure S1J).

### The stress-induced sleep-promoting ALA neuron is required for quiescence after training and long-lasting memory

The two sleep-promoting neurons, ALA and RIS, regulate different types of sleep.^23^ ALA is required for stress-induced sleep,^27,58,60^ whereas RIS is required for developmentally timed sleep.^61^ Therefore, we asked if *ceh-17(np1)* mutants, which have defective ALA neurons and cannot promote stress-induced sleep,^62,63^ become movement quiescent after training. Although naive *ceh-17* mutants and wild-type animals show similar movement, *ceh-17* mutants are less quiescent than wild types after butanone training (Figure 1L). This indicates that quiescence after training requires ALA. Further, although *ceh-17* mutants learned equally well as wild-type animals, their memory is disrupted 16 h later (Figure 1M). In contrast, RIS-defective *aptf-1* mutants were able to learn and remember similar to wild-type animals (Figure 1M). This indicates that ALA, but not RIS, is necessary for post-training quiescence and odor memory, which is consistent with the post-training state being a form of stress-induced sleep. Swimming is energetically costly^64^ and can induce sleep,^28^ and it is possible that during training this induces sleep.

The adenylyl cyclase *acy-1* acts downstream of ALA, and *acy-1(ce2)* mutants are defective for stress-induced sleep.^23^ We found that *acy-1(ce2)* mutants neither enter a sleep state after training nor remember 16 h later (Figures S3A-S3C). Given the dependence of the post-training quiescent state on the ALA neuron, its disruption in *acy-1(ce2)* mutants, and its shared sleep characteristics, we consider the post-training quiescent state to be a sleep state.

### CREB is required for long-term memory

**Figure 2.**
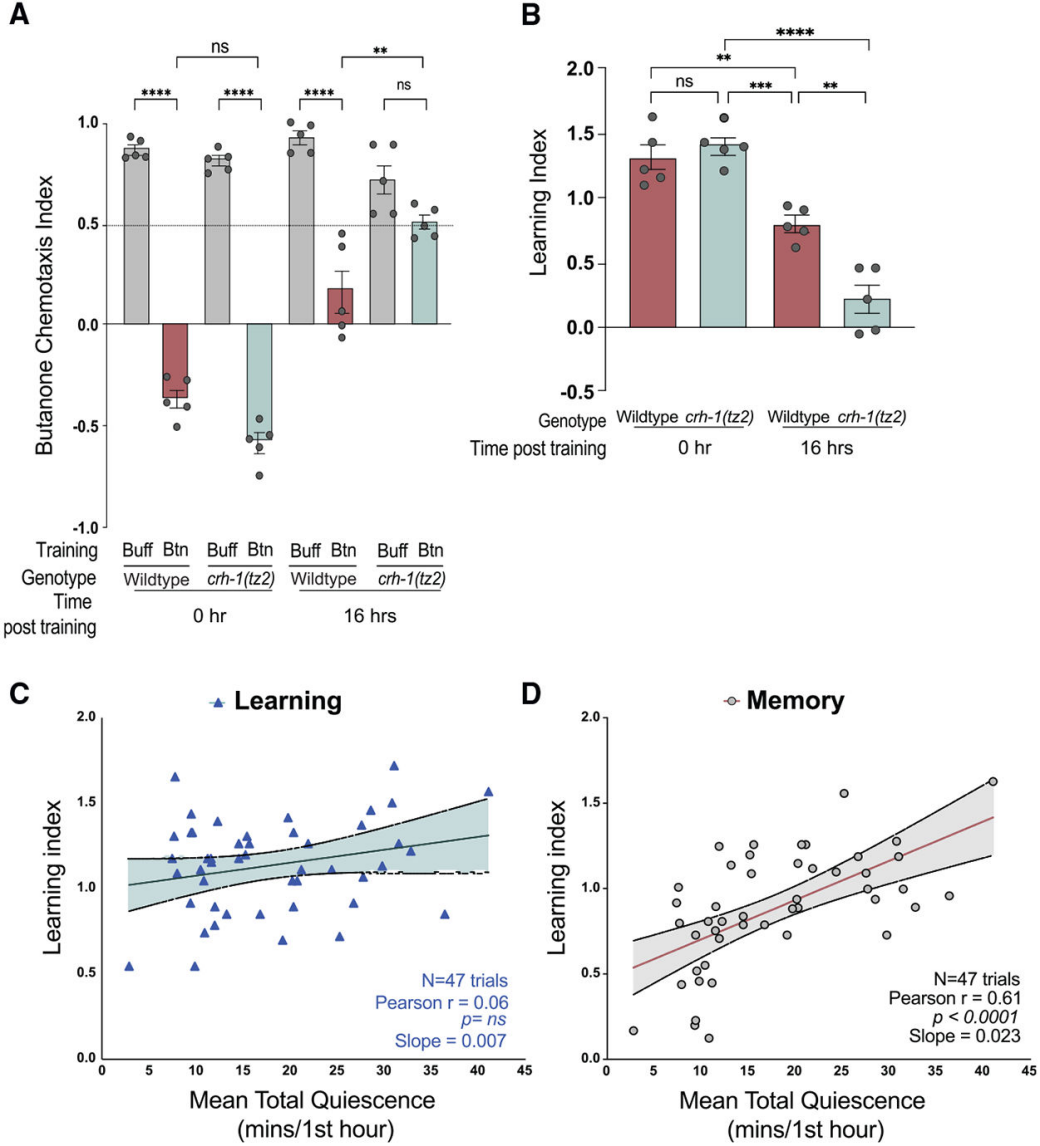
Long-lasting olfactory memory requires CREB and is correlated with quiescence after training (A and B) (A) CIs and (B) LIs for wild-type animals and CREB-defective crh-1(tz2)/CREB mutants immediately and 16 h after training (****p < 0.0001, ***p < 0.001, **p < 0.005 and (ns) is p > 0.05, one-way ANOVA with Bonferroni’s multiple correction, n = 5 trials. All error bars are ±SEM. (C) The LI of each population at t = 0 (before recovery) is plotted versus the mean duration of quiescence in the hour after training. n = 47 trials, Pearson’s correlation coefficient is 0.06 p = ns. (D) The LI at t = 16 (after recovery) is plotted versus the mean duration of quiescence in the hour after training. n = 47 trials, Pearson’s correlation coefficient (r) is 0.61, p < 0.0001. Table S1 supports the statistics and Table S2 contains the raw and analyzed data for this figure.

The CREB has a conserved role in long-term memory^50,65,66^; therefore, we asked if it plays a role in this olfactory learning paradigm. *crh-1(tz2)/CREB* mutants learn as well as wild types but fail to keep the memory after 16 h (Figures 2A and 2B). Thus, the long-term memory, but not initial learning, is likely to be CREB transcription dependent.

### Sleep duration after training strongly correlates with memory

Although post-training sleep has been shown to benefit memory formation in other systems,^66^ whether the amount of sleep correlates directly with increased learning or memory is unknown in *C. elegans*. We found that the amount a population learns is not correlated with sleep duration in the first hour after training (Figure 2C). However, memory 16 h after training strongly correlates with the amount of sleep a population exhibits in the hour immediately after training (Figure 2D). Thus, the more a population slept after training, the better the memory consolidation.

### Sleep is necessary for long-term memory

**Figure 3.**
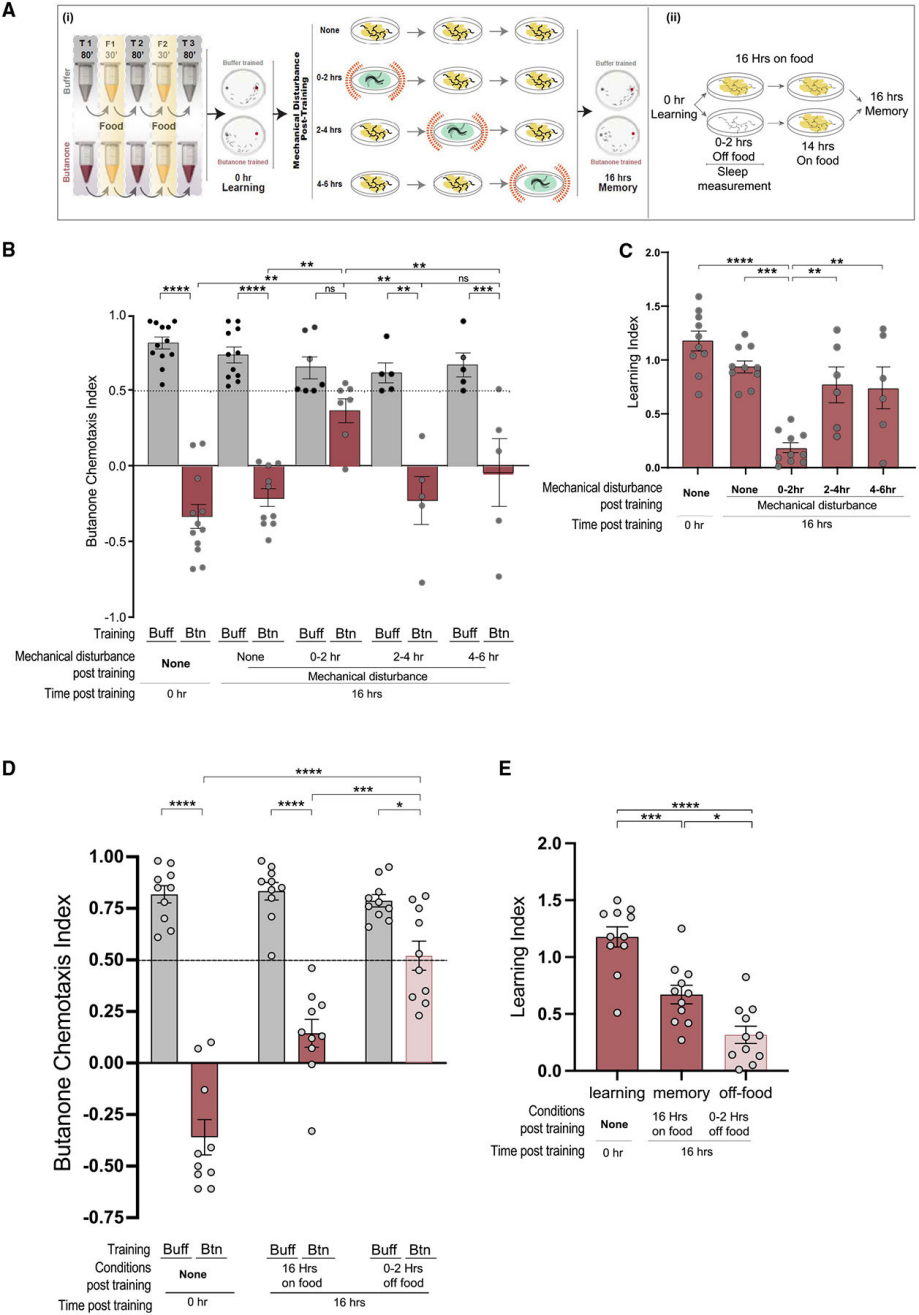
Disturbing animals immediately after training blocks memory (A) (i) Mechanical disturbance: training is followed by shaking (red springs) animals in lower viscosity food every 15 min for 2 h from 0–2, 2–4, or 4–6 h after training. Figure S3 indicates that animals eat during mechanical disturbance. Videos of sleep disruption are in Video S3, related to Figure 2. After disturbance, animals are recovered on food without shaking until 16 h after training. (ii) Metabolic disturbance: animals are recovered on agar petri dishes without bacteria for 2 h immediately after training, then moved to bacterial lawns for 14 h. Quiescence was measured during the period off food. (B) CIs of mechanically disturbed populations. ****p < 0.0001, ***p < 0.001, **p < 0.01, *p < 0.05, and (ns) is p > 0.05, two-way ANOVA with Bonferroni’s multiple correction, n = 5 trials. (C) LIs of mechanically disturbed populations. ****p < 0.0001, ***p < 0.001, **p < 0.01, and (ns) is p > 0.05, one-way ANOVA with Bonferroni’s multiple correction, n < 5 trials. (D and E) (D) CIs and (E) LIs of animals removed from food 0–2 h after training. (****p < 0.0001, ***p < 0.001, **p < 0.01, *p < 0.05, and (ns) is p > 0.05, one-way ANOVA with Bonferroni’s multiple correction, n > 5 trials. All error bars are ±SEM. Figure S4 and Table S1 supports the statistics and Table S2 contains the raw and analyzed data for this figure.

We asked whether *C. elegans* requires post-training sleep to consolidate memory. Mechanically disrupting sleep in the first 2 h after training (Figure 3A) blocked quiescence (Video S3) and memory retention (Figures 3B and 3C). Importantly, animals ate the same amount whether or not they were mechanically disturbed (Figure S4A). By contrast, cohorts that were disturbed 2–4 or 4–6 h after training kept the memory (Figures 3B and 3C). This indicates that sleep in the first 2 h after training is critical for memory, but is not required later. Sleep can also be disturbed by removing *C. elegans* from their bacterial food source, causing them to roam.^42,67,68^ Removal from food for the first 2 h after training blocked sleep (Video S2; Figure S4F) and memory (Figures 3E and 3F). Thus, two experimental paradigms that disrupt sleep immediately after training interfere with long-term memory. To our knowledge, this is the first example of sleep being required for memory consolidation in an organism with such a reduced nervous system.

### Sleep enhances long-term memory of butanone

**Figure 4.**
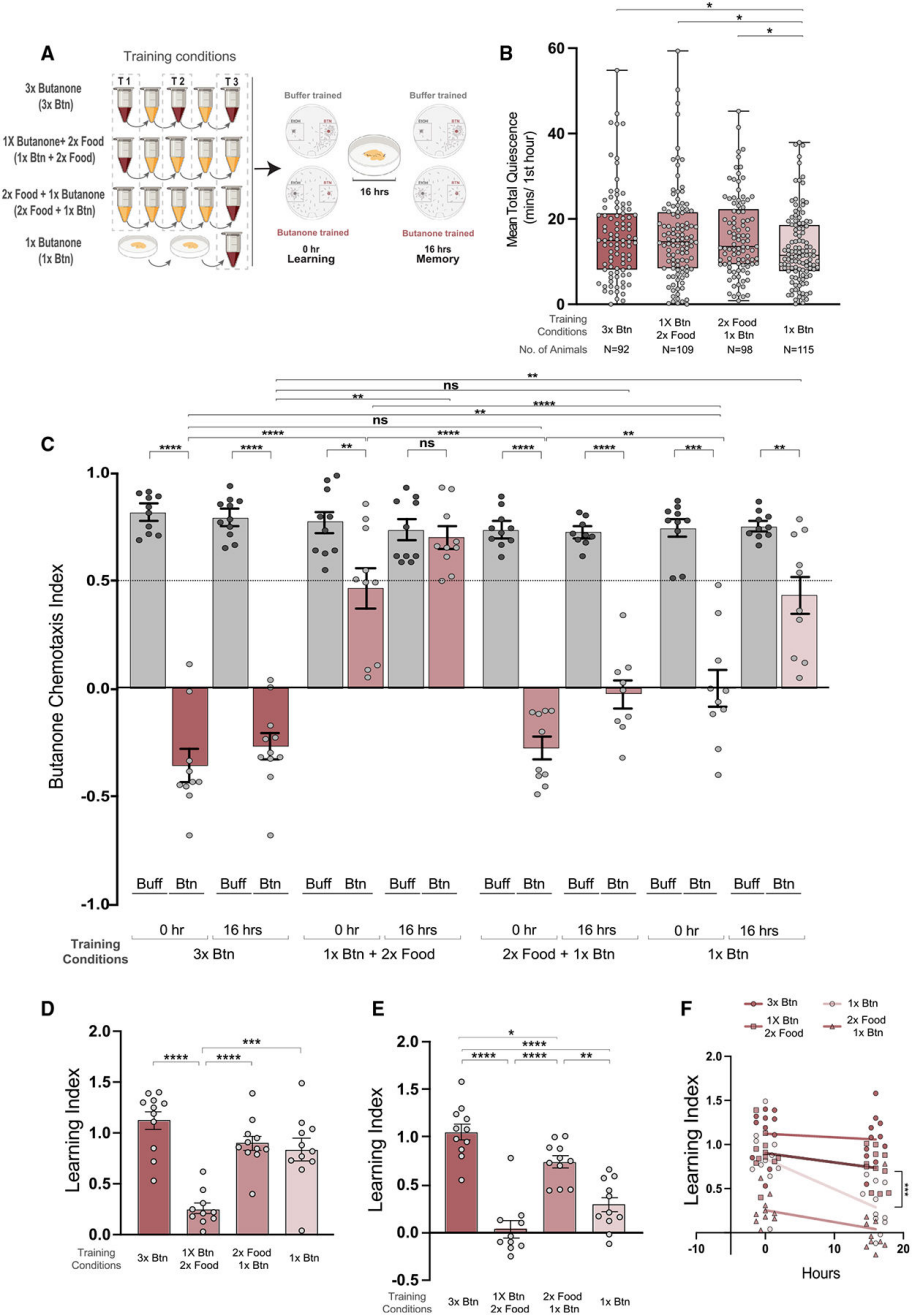
Increasing sleep increases memory (A) Animals were trained for three cycles with butanone (top row, 3XBtn), trained once with butanone then swam in food mixed with buffer for two cycles (1XBtn + 2XFood), swam in food for two cycles then trained once with butanone (2XFood + 1XBtn), or trained once with butanone (bottom row, 1XBtn). After training, quiescence was assessed with WorMotel. Learning was assessed with a chemotaxis assay immediately after training and memory with a chemotaxis assay 16 h later. (B) Mean total quiescence was determined after each training paradigm. p < 0.05, unpaired t test with Welch’s correction, n = 5 trials. (C) CIs of populations after each training paradigmat 0 or 16 h post-training. ****p < 0.0001, ***p < 0.001, ** p < 0.01, *p < 0.05, and (ns) is p > 0.05, two-way ANOVA with Bonferroni’s multiple correction, n = 10 trials. (D) LIs immediately after training. (E) LIs 16 h after training. ****p < 0.0001, ***p < 0.001, **p < 0.01, *p < 0.05, and (ns) is p > 0.05, one-way ANOVA with Bonferroni’s multiple correction,n = 10 trials. (F) ***p < 0.001, comparison of slopes using linear regression shows the amount of memory lost between 0 and 16 h for each training condition. All error bars are ±SEM. Table S1 supports the statistics and Table S2 contains the raw and analyzed data for this figure. See also Figure S5.

We asked if increasing sleep is sufficient to convert a weak, short-term memory into a long-term one (Figure 4A). After only one cycle of training, animals exhibit little post-training sleep (Figure 4B). They learn to ignore butanone (Figures 4C and 4D), but have lost the memory 16 h later (Figures 4C and 4E; Benedetti et al.^69^). Sleep is increased if animals swim in liquid food before the odor training cycle (Figures 4A and 4B), and memory 16 h post-training is significantly increased when compared with animals that had equivalent odor training, but less sleep (Figures 4C and 4E). Cohorts trained with one cycle of butanone followed by swimming in food showed poor learning and memory (Figures 4C-4E). This could result from lack of sleep after training. We also found that memory decayed less with increased sleep (Figure 4F).

### Long-term memory does not accompany changes in AWC sensory neuron activity

**Figure 5.**
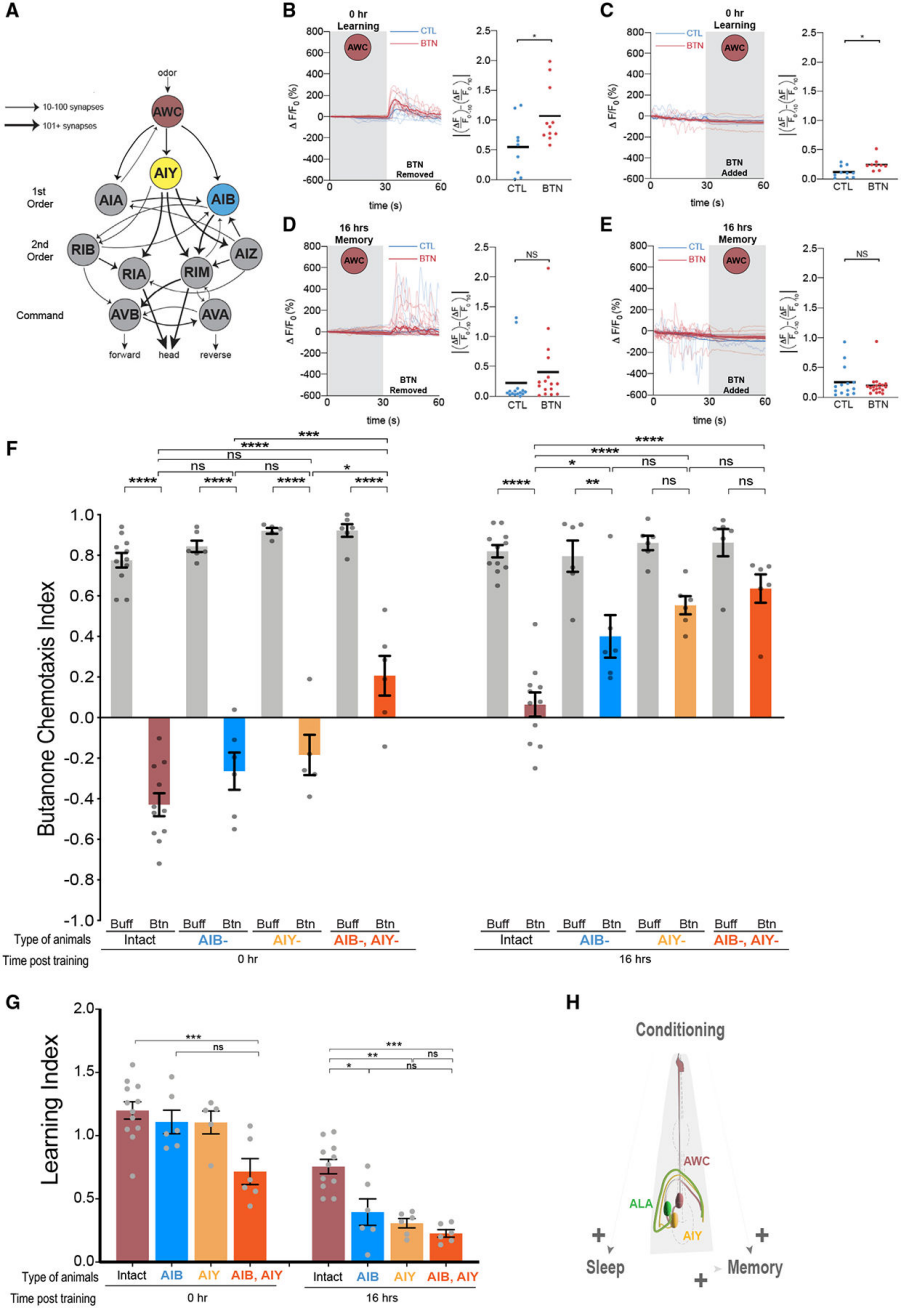
The interneuron AIY is required for sleep-dependent memory (A) Diagram of the AWC olfactory circuit. AWC sensory neurons (red) are inhibited by odor. Smallest arrow indicates 1–10 chemical synapses, medium arrow, 10–100 synapses, and largest arrow, more than 100 synapses, (gap junctions not shown). Figure adapted from Gordus et al.^43^ (B–E) Calcium transients were visualized using GCaMP3) in the AWC^ON^ of a trapped animal as it is exposed to butanone (gray shaded area) or buffer (white). Blue traces are transients in control-trained and red traces are those of butanone-trained animals. Plots show the change in fluorescence immediately before and after the change in stimulus and each point signifies one worm. ns p > 0.05, *p < 0.05, paired t test. (B and C) Transients measured immediately or (D and E) 16 h after training. (F and G) (F) The CIs and (G) LIs of animals missing no neurons (brick), AIBs (blue), AIYs (yellow) or both AIBs and AIYs (orange) immediately (t = 0) or after 16 h after training. ****p < 0.0001, ***p < 0.001, **p < 0.01, *p < 0.05, and ns p > 0.05, one-way ANOVA with Bonferroni’s correction, n > 5 trials. (H) Model: spaced olfactory conditioning induces sleep and memory in *C. elegans*. Sleep induced by butanone conditioning is ALA-dependent and benefits memory retention. The signal that butanone has been sensed passes from the AWC neuron to interneurons including AIY and AIB. Memory requires AIY, thus we hypothesize that sleep may act on connections between AWC and AIY neurons. All errors are SEM. Table S1 supports the statistics and Table S2 contains the raw and analyzed data for this figure. See also Figure S5.

We asked whether changes in the AWC sensory neuron response underlie the observed memory. To monitor calcium transients (Figure 5A), we imaged animals expressing the GCaMP3 calcium indicator in AWC^ON70^ immediately after conditioning and again 16 h later (Figures 5B-5E). AWC calcium levels have been found to decrease when animals are exposed to butanone, rise after odor removal, then return to baseline.^40,46^ We find that odor removal triggers a small but significantly higher calcium increase in AWC immediately after butanone-training, compared with buffer-training (Figure 5B). Likewise, odor onset triggers a small, but significantly greater silencing of AWC in butanone-trained animals compared with the buffer-trained controls (Figure 5C). However, 16 h later, the differences in the responses between buffer- and butanone-trained animals disappear (Figures 5D and 5E). Thus, it is unlikely that a change in the AWC sensory response promotes long-term memory.

### The long-lasting memory requires AIY post-synaptic interneurons

We reasoned that the cells responsible for memory might be downstream of AWCs in the chemosensory circuit. Serial electron micrographs indicate that the AWCs form synapses with three pairs of interneurons: the AIYs, AIBs, and AIAs.^15,18,71^ Because AIAs are required for butanone learning (Cho et al.^46^), learning defects of AIA-ablated strains were not assessed.

We found that animals lacking AIYs (*ttx-3* cell-fate mutants^72^), or AIBs(caspase-killed^73,74^), exhibit normal chemotaxis and learning, although doubly ablated animals have learning defects (Figures 5F and 5G). Animals lacking AIY interneurons have more consistent memory loss 16 h post-training than those that lack AIB, and they retain a similar amount of memory as AIY and AIB doubly ablated animals (Figures 5F and 5G). AIY ablated animals also lose more memory over the 16 h after training (Figure S5). Thus, we focused on understanding whether sleep affects connections between the AWC and AIY neurons (Figure 5H).

### AWC-AIY synapses are visualized with NLG-1 GRASP

**Figure 6.**
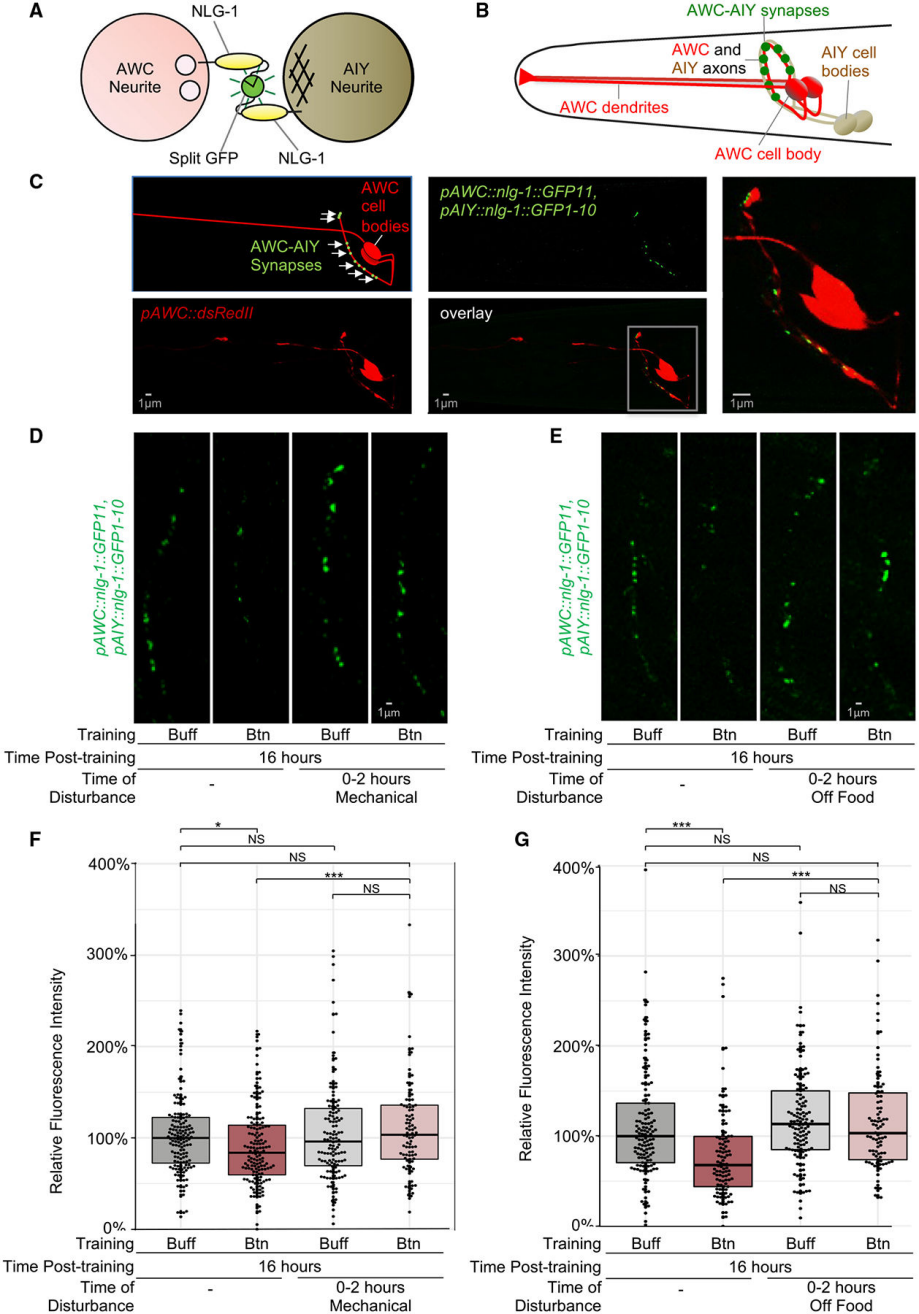
Odor training and sleep result in AWC-AIY synaptic reductions (A) Schematic of split GFP-based AWC-AIY NLG-1 GRASP marker. Circles represent cross-sections of the AWC and AIY neurites, and one neurite from each neuron pair is represented for simplicity. Split GFP fragments are linked to the pre- and post-synaptically localized protein NLG-1 (neuroligin 1) and expressed in the AWC and AIY neurons with the selective promoters *_p_odr-1* and *_p_ttx-3*. When synapses form between the neurons, the split GFPs come in contact, reconstitute, and fluoresce.White circles indicate a presynaptic site, and crosshatching represents a post-synaptic site. (B) Schematic of the head of an animal in which NLG-1 GRASP labels synapses between the AWC (red) and AIY (beige) neurites in the nerve ring, which forms an arch in the head of the animal. (C) Schematic and micrographs of an animal carrying the AWC-AIY NLG-1 GRASP marker with the AWC neurons labeled in red with the cytosolic mCherry fluorophore. Synaptic fluorescence is observed in a punctate pattern in AWC axons in the nerve ring. The area in the gray box is expanded in the rightmost image. (D) Micrographs of AWC-AIY NLG-1 GRASP fluorescence 16 h after training with control buffer (Buff) or butanone (Btn) in which sleep was not disrupted after training (left two micrographs), and in which sleep was disrupted by mechanical disturbance for the first 2 h after training (right two micrographs). (E) Micrographs of AWC-AIY NLG-1 GRASP fluorescence 16 h after training with control buffer or butanone in which sleep was not disrupted after training (left two micrographs), and in which sleep was disrupted by removal from food for the first 2 h after training (right two micrographs). (C–E) Scale bars are 1 micron. (F) Quantification of the reduction in AWC-AIY NLG-1 GRASP fluorescence intensity in animals trained with butanone whose sleep was not disrupted, in comparison with animals whose sleep was disrupted by mechanical disturbance and animals trained with control buffer. (G) Quantification of the reduction in AWC-AIY NLG-1 GRASP fluorescence intensity in animals trained with butanone whose sleep was not disrupted, in comparison with animals whose sleep was disrupted by removal from food and animals trained with control buffer. (F and G) Animals were imaged were from populations of buffer-trained or sleep-deprived animals that chemotaxed to butanone (CI > 0.5) or butanone-trained populations allowed to sleep that did not chemotaxis to butanone (CI < 0.5). n > 90 for each box and includes animals trained on four different days. NS p > 0.05, *p < 0.05, ***p < 0.001, Mann-Whitney U test. p values were adjusted for multiple comparisons using the Hochberg procedure. Figure S6 and Tables S3 and S4 support this figure.

To understand the mechanism by which memory is stored, we asked if olfactory synapses are altered in animals that remember their training. We examined synaptic connections from AWC chemosensory neurons into AIY interneurons, because AIY neurons are more important for olfactory memory consolidation (Figures 5F, 5G, and S5G), and AWCs for the most synapses with AIYs.^18^ To visualize AWC-AIY synapses, we utilized NLG-1 GRASP, a split GFP-based transsynaptic marker (Figure 6A^49,75,76^). Two complementary GFP fragments, GFP1-10 and GFP11,^77^ are linked to NLG-1, which localizes to pre- and post-synaptic sites in *C. elegans* and colocalizes with synaptic markers.^49^ When synapses form, the split-GFP fragments reconstitute and fluoresce (Figure 5A). NLG-1 GRASP synaptic patterns are consistent with electron microscopy reconstruction data for many different neuron pairs; green fluorescent puncta are observed in regions known to form synapses between the neurons of interest, whereas regions without synapses are not labeled.^15,18,49,71,75,76^

Electron micrograph reconstruction studies indicate that AWC neurons form en passant synapses onto the left and right AIY neurons in the nerve ring.^15,18^ To visualize AWC-AIY synapses, we drove expression of NLG-1::GFP11 and NLG-1::GFP1-10 in AWC and AIY neurons, respectively. We found that AWC-AIY NLG-1 GRASP labeling results in fluorescent green puncta along the AWC axons in the nerve ring (Figures 6B and 6C). The localization and distribution of AWC-AIY NLG-1 GRASP fluorescent puncta along the nerve ring in the head (Figure 6C) was consistent with previous electron micrograph reconstructions.^15,18,49,71,75,76^

### AWC-AIY synapses are reduced in animals with the olfactory memory

To determine if AWC-AIY synapses are physically altered in animals that retain the olfactory memory, we imaged AWC-AIY NLG-1 GRASP-carrying odor-trained animals with the memory and buffer-trained control animals without the memory. Interestingly, we found that AWC-AIY NLG-1 GRASP fluorescence intensity was significantly reduced in butanone-trained populations that held the olfactory memory, when compared with buffer-trained controls (Figures 6D, 6E, S5A, and S5B). We quantified AWC-AIY NLG-1 GRASP intensity in these populations and found that the synaptic signal in butanone-trained animals was significantly lower than in control buffer-trained animals (Figures 6F, 6G, S5A, and S6B). Thus, training with butanone results in a synaptic reduction between chemosensory neurons and the post-synaptic cells that are required for olfactory memory. We found that AWC-AIY synaptic intensity was similar immediately before training and 16 h after buffer-training (Figure S6C), indicating that synapses are reduced in butanone-trained animals.

### Sleep is required for AWC-AIY synaptic reductions after odor training

We asked if the 2 h of post-training sleep needed for olfactory memory is also required for the AWC-AIY synaptic reductions that we observed in butanone-trained animals 16 h after training. We disrupted sleep by either shaking the worm plates every 15 min (as in Figure 3A) or removing the animals from food for 2 h immediately after training (as in Figure 3D). Animals were then moved to food plates for 14 h before assessing synapses. We found that animals whose sleep was disrupted immediately after training and that lost olfactory memory lacked synaptic reductions (Figures 6D-6G, S5A, and S5B). Further, these butanone-trained animals that were deprived of sleep had significantly higher synaptic intensity than their butanone-trained counterparts that slept after training, and not significantly different from that of buffer-trained control animals (Figures 6D-6G, S5A, and S5B). These data indicate that post-training sleep is required for butanone training-induced AWC-AIY synaptic reductions, which correlate with memory. Furthermore, 16 h after training, synapse levels correspond with behavioral responses: lower AWC-AIY synaptic levels are found in populations with weaker attraction to butanone, whereas higher synaptic levels are found in populations with a stronger attraction to the odor (Figures 6D-6G, S5A, and S5B).

To determine if synaptic changes in response to butanone training are global, we examined connections between PHB chemosensory neurons and two primary post-synaptic partners, the AVA interneurons. PHBs sense noxious chemicals, including dodecanoic acid^78^ and sodium dodecyl sulfate.^79^ Using a strain that carries PHB-AVA NLG-1 GRASP,^75,76^ we found that PHB-AVA synapses are not significantly altered by butanone-training or sleep (Figures S6D-S6F). This indicates that the synaptic changes induced by butanone training and sleep are not global.

### AWC-AIY synapses are reduced in odor-independent and odor-dependent manners

**Figure 7.**
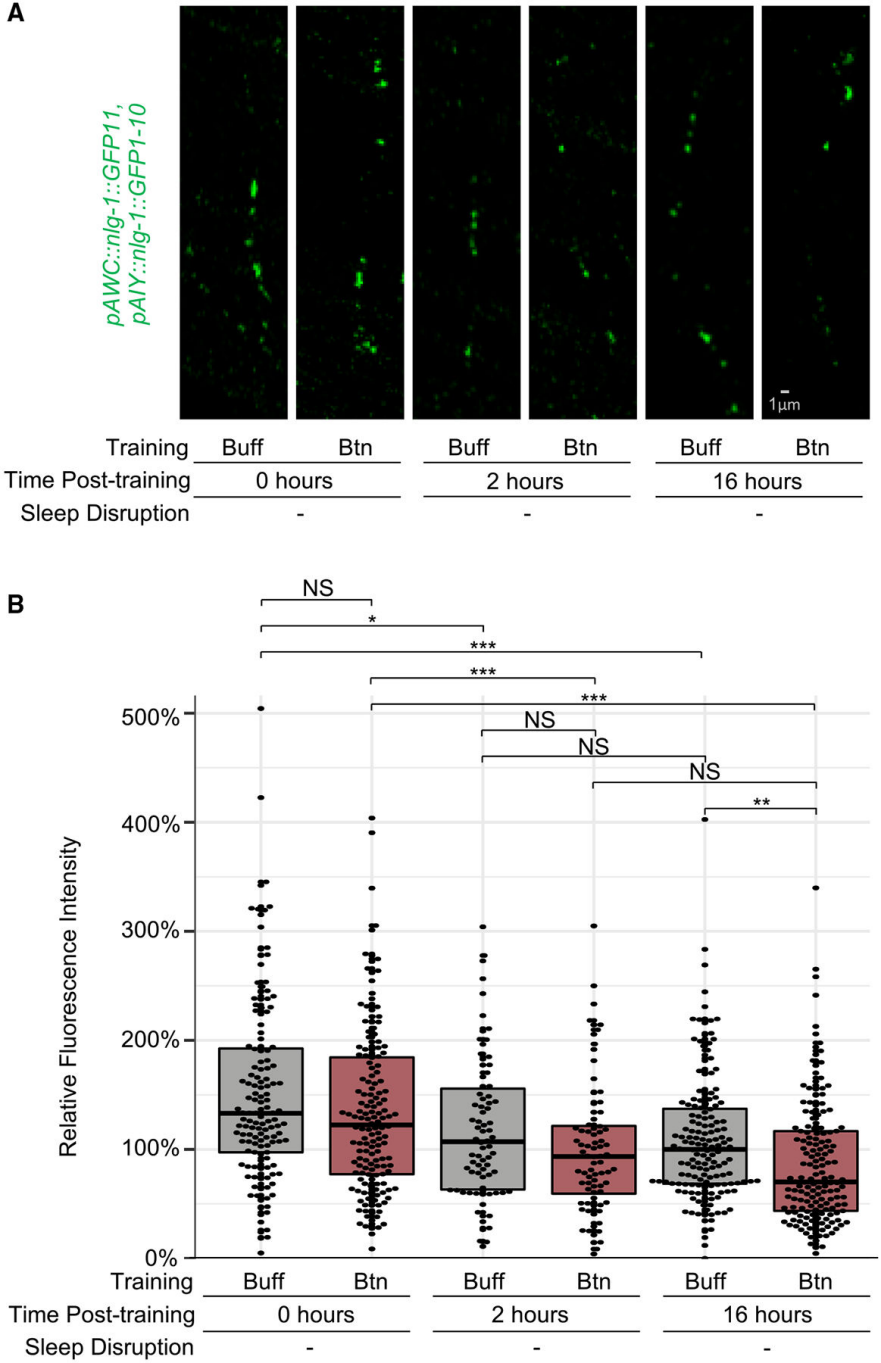
AWC-AIY synapses are altered during and after sleep (A) Micrographs of AWC-AIY NLG-1 GRASP fluorescence in animals trained with butanone (Btn) or control buffer (Buff) at 0, 2, and 16 h after training without sleep disruption. Scale bar is 1 micron. (B) Quantification of AWC-AIY NLG-1 GRASP fluorescence intensity at 0, 2, and 16 h post-training in buffer-trained and butanone-trained animals whose sleep was not disrupted. N > 75 for each box and includes animals trained on four different days. NS p > 0.05, *p < 0.05, **p < 0.01 and ***p < 0.001, Mann-Whitney U test. p values were adjusted for multiple comparisons using the Hochberg procedure. Animals were imaged were from populations of buffer-trained animals that chemotaxed to butanone (CI > 0.5) or butanone-trained populations that did not chemotaxis to butanone (CI < 0.5) (Figure S7F). Figure S7 and Tables S3 and S4 support this figure.

To understand whether AWC-AIY synapses change during sleep after training, we examined AWC-AIY synapses in populations of animals immediately after training and after the 2-h period of post-training sleep. We found that immediately after training, both control buffer-trained (CI > 0.5) and butanone-trained animals (CI < 0.5) begin with higher synaptic levels than in buffer-trained animals 16 h post-training (Figures 7A and 7B). This indicates that training with butanone alone does not instantly alter synaptic structures. However, during the 2-h critical period of sleep after training, synapses in both buffer- and butanone-trained worms are similarly reduced (Figures 7A and 7B), consistent with our observations in single animals (Figure S7). This indicates that during the first 2 h after training, synapses are reduced in an odor-training-independent manner.

To understand how olfactory synapses change during the following 14 h, we analyzed synaptic intensity in animals from the 16-h time point of population time course assays. We observed a significant reduction of AWC-AIY synapses when compared with control buffer-trained animals (Figures 7A and 7B), similar to that observed in previous assays (Figures 6D-6G). This indicates that, although synaptic levels are similar in buffer- and butanone-trained animals 2 h after training, they become distinct in the two populations 16 h after training.

To test whether the rate of synaptic change was significantly different between 0–2 and 2–16 h after training for butanone-trained and buffer-trained animals (separately), we fitted a continuous, piece-wise-linear spline least squares regression model to the square-root-transformed response separately for butanone- and buffer-trained animals as a function of time. The square-root transformation was necessary to satisfy the model assumption of normally distributed residuals. For this spline function, one can test whether the slope between 0–2 h and the slope between 2–16 h is significantly different. In both cases the slope differs significantly (p value is 0.00410 for buffer-trained and 0.00175 for butanone-trained animals). This indicates that the rate of synaptic change in the first 2 h after training is significantly different than the rate of synaptic change in the following 14 h. Thus, we observe a rapid odor-training-in-dependent synaptic reduction during the first 2 h post-training, followed by a period of slower synaptic change in the following 14 h during which synaptic levels in butanone-trained animals become lower than those in buffer-trained animals. Although this second period of synaptic reduction requires sleep after training (Figures 6D-6G), structural changes are not complete until after the critical period (Figure 7).

## DISCUSSION

Our results provide several insights into how specific memories are strengthened by sleep. First, we discovered that *C. elegans* require a quiescent period immediately after training for long-term CREB-dependent olfactory memory. This quiescence has the behavioral hallmarks of sleep and requires the ALA sleep neuron. The finding that *C. elegans*, with only 302 neurons, requires sleep to consolidate memory reveals that it is not the complexity of the nervous system that demands sleep, but another aspect shared by the simplest of nervous systems. We next found that sleep is required immediately after training. Finally, we found that the structural connections between an olfactory synaptic partner pair that is required for memory are reduced in a sleep- and training-dependent manner. This indicates that nervous system complexity is not required for sleep-dependent synaptic plasticity. Our results indicate that, although the effects of sleep are global, it can specifically affect long-term memory formation and olfactory circuit modulation.

### Sleep characteristics of the post-training quiescent period

During training, exercise and re-feeding likely mobilizes fat stores, which induces quiescence.^28,67,68^ During the post-training quiescent period, we show that most hallmarks of sleep are similar to those previously described in the literature: movement quiescence (Figure 1D),^21,57^ arousal latency (Figures 1H and 1I),^59^ and posture (Figures 1E and S2C).^22,56,57^ However, although pumping was significantly reduced (Figure 1J), it was not reduced as much as in heat-shocked animals (Figure S1J^58^). The synchronicity of reduced feeding in heat-shocked animals may result from the acuteness of the perturbation.

In addition, disruption of the stress-induced sleep-promoting ALA neuron, but not the developmental-timed sleep-promoting RIS neuron,^23^ significantly reduces mobility quiescence after training (Figure 1L) and interferes with long-lasting memory (Figure 1M). Nath and colleagues showed that specific sleep peptides secreted from the ALA neuron are responsible for immobilizing specific organs.^27^ During learning and memory, it is possible that a subset of these may be employed to instruct the sleep characteristics that we observe.

### Learning, sleep, and memory consolidation modulate the olfactory circuit

In animals with consolidated memory, the glutamatergic odor-OFF AWC^ON^ olfactory neuron shows responses to butanone that are identical to those of buffer-trained controls (Figures 5D and 5E). This implicates changes in the circuit downstream of the sensory neuron, including AIY, AIB, and AIA interneurons.^18,42,43^ *C. elegans* approach AWC-sensed odors with a combination of smooth, curved runs up the odor gradient called klinotaxis^16,80,81^ that may depend on AIYs and their post-synaptic partners, the RIA interneurons,^82-84^ and turn-driven reorientations called pirouettes^85^ that depend on activation of AIBs when the gradient decreases.^42,43,46^ Moving down an odor gradient results in a combination of corrections initiated by increased AWC signaling.^40,86^

Although the effect of sleep-dependent memory on odor gradient navigation has not been examined, initial sleep-independent learning^87^ and memory after longer, 10-h blocks of training paired with starvation^88^ have been investigated. The decrease in butanone responsiveness immediately after training likely depends on transcriptional changes in AWC,^45,89,90^ some of which are mediated by insulin signaling that reduces butanone-evoked glutamate release.^87^ When animals descend the gradient, this could result in less inhibition of AIY interneurons, permitting runs away from butanone. Likewise, diminished activation of AIB interneurons^46^ could inhibit reorientation. This could explain the impaired chemotaxis immediately after training in our paradigm before synaptic structures are modulated.

Although we were unable to assess AIA’s role in sleep-dependent memory due to its learning defect,^46,87^ Pritz and colleagues studied animals that exhibit long-term memory after prolonged (10 h) starvation. They observed reduced overall speed and increased turns while navigating a butanone gradient, which they posit reflects increased AIA activity.^88^

This diminished AWC neurotransmitter release immediately after training^87^ when coupled with post-training sleep may lead to the reduction of AWC-AIY synaptic structures we observe hours later. This may transform the transient post-training synaptic modulation into a more permanent change in synaptic structures. Overall, AWC sensory input should be less coupled to downstream AIY interneurons, reducing the ability to reach an odor source.

### Synaptic reductions could contribute to memory consolidation

2 h after training, the synapses between the AWC and AIY neurons are equivalently reduced in buffer- and butanone-trained animals. This is reminiscent of the widespread synaptic downscaling observed in complex systems during sleep, which is posited to reset the brain by reducing global synaptic strength.^36^ However, observations of synapses throughout the animal will be necessary understand if sleep in *C. elegans* also produces global downscaling.

16 h after training, AWC-AIY synapses are reduced in butanone-trained compared with buffer-trained animals. This odor-dependent synaptic reduction shares characteristics with synaptic consolidation,^91^ in which changes in synaptic activity immediately after learning transition to more permanent structural changes in synaptic architecture. Likewise, reduced glutamate release from AWCs immediately after training^87^ likely precede the structural changes we observe in animals with consolidated memory. Systems consolidation describes the apparent movement of a memory trace from the site of learning to distinct regions within the brain for long-term storage.^37^ The finding that AIY interneurons are required for memory, but not learning, and that their synapses with AWC chemosensory neurons are reduced with respect to control buffer-trained animals 16 h after, but not immediately after training, may also support the idea that a kind of systems consolidation process is initiated during post-training sleep.

Our work also indicates that a tight temporal link between odor training and sleep is critical for memory and synaptic reduction at 16 h. This suggests that odor-training-induced changes may mark synapses for reduction and may need to be immediately acted on by sleep to promote long-lasting changes. A temporal link between training and sleep has also been demonstrated to be important for memory in vertebrates.^29,92^

In studies of learning, examples of synaptic increases abound. However, synaptic reductions in learning have also been documented.^93^ Indeed, extinction paradigms in which animals are trained to forget a learned behavior exhibit synapse reduction and long-term depression.^93^ Although our paradigm may instead engage inhibitory operant conditioning, it is tempting to speculate that synaptic reduction may be an important component of learning and remembering. Further, synaptic reductions may drive learning in motor coordination tasks, in which it is as important to not contract unnecessary muscles as it is to contract the correct ones. For example, REM sleep has been shown to both reduce and strengthen synapses in layer 5 of mouse cortex after motor learning.^94^

### Long-term olfactory memory decay

Forgetting can occur after learning, such that memory is significantly reduced within 2 h of training.^95-97^ The memory described in our work is kept for at least 24 h if the animals are allowed to sleep after training, indicating that forgetting may be blunted by sleep. The impact of sleep on forgetting will be an interesting avenue for future studies.

Extinction is functionally similar to forgetting but requires exposure to the unconditioned stimulus in the absence of the conditioned stimulus after training. The butanone training paradigm resembles inhibitory operant conditioning in which a voluntary behavior is inhibited by punishment. Inhibitory operant conditioned memory has been shown to require sleep in invertebrates and vertebrates.^98,99^ Interestingly, the extinction of inhibitory operant conditioned memory is promoted by wakefulness in the presence of the unconditioned stimulus.^9^ However, we found that animals that were maintained on food after conditioning, and whose sleep was mechanically disrupted so that they were not re-exposed to the unconditioned stimulus, lost the memory 16 h after training. This indicates that the loss of the butanone long-term memory was due to loss of sleep, rather than extinction.

### Limitations of the study

Although olfactory training results in a state that shares many characteristics of sleep, including increased movement quiescence, stereotypical posture, increased arousal latency, and reduced feeding, the percentage of the population that completely ceases feeding is not as large as that observed during heat-shock-induced sleep (Hill et al.^58^; Figure S1J). Similar to animals exhibiting forced swimming fatigue, pumping does not cease for all animals at once.^100^ Unlike forced swimming fatigue, odor-training-induced quiescence requires the ALA sleep neuron, which is also required for stress-induced sleep^23^ and makes us favor the interpretation that this is a sleep state. We suggest that, although training may trigger stress-induced ALA-dependent sleep, the animals still pump (albeit more slowly) because they are in food, which causes serotonin release.^101^ Serotonin has been shown to promote pumping in animals in stress-induced sleep,^23^ and this may be what we see in our trained populations once they are returned to a lawn of food.

## STAR★METHODS

### RESOURCE AVAILABILITY

#### Lead contact

Further information and requests for resources and reagents should be directed to and will be fulfilled by the lead contact: Noelle L’Etoile (noelle.letoile@ucsf.edu).

#### Materials availability

Plasmids, *C. elegans* lines and datasets generated in this study are available on request from the lead contact.

#### Data and code availability

- All data displayed in figures is available in Tables S2 and S4. Microscopy data reported in this paper will be shared by the lead contact upon request.
- All original code has been deposited at GitHib and is publicly available as of the date of publication
- Any additional information required to reanalyze the data reported in this paper is available from the lead contact upon request and in Table S2.

### EXPERIMENTAL MODEL AND SUBJECT DETAILS

#### *C. elegans* strain cultivation

All *C. elegans* worms were reared according to standard protocols.^104^ All strains were raised at 20°C or room temperature and tested at room at temperature. Animals were raised on 10cm Nematode Growth Media (NGM) plates with an OP50 *E. coli* lawn. All assays were started when animals were day one gravid adults. All strains used in this study are listed in the key resources table. The JZ2008 AIB and AIY double kill strain *(ttx-3(ks5) X; peIs578 (_p_npr-9::casp1; _p_unc-122::mCherry; _p_npr-9::venus)* was made by mating the *FK134/ttx-3(ks5)* X strain with the *peIs578 (_p_npr-9::casp1; _p_unc-122::mCherry; _p_npr-9::venus)* strain from the CGC and the Iino lab, respectively. Other transgenic strains generated for this study are *iyIs35 (_p_ttx-3::nlg-1::GFP1-10*^49^ (70ng/μl), *_p_odr-1::nlg-1::GFP11* (see below for generation) (40ng/μl), *pOdr-1::DsRedII*^102^ (5ng/μl) and *_p_unc-122::RFP*^103^ (20ng/μl)) and *wyIs155 (_p_gpa-6::nlg-1::GFP1-10* (Park et al., 2011) (60 ng/μl), *_p_flp-18::nlg-1::GFP11*^75^ (30 ng/μl), *_p_nlp-1::mCherry* (Park et al., 2011) (10 ng/μl), *_p_flp-18::mCherry* (Park et al., 2011) (5 ng/μl) and *_p_odr-1::DsRedII* (20 ng/μl)). Constructs were generated using standard molecular techniques. To generate *_p_odr-1::nlg-1::GFP11*, the *odr-1* promoter was amplified from *_p_odr-1::DsRedII*^102^ using *_p_odr-1*-specific primers (MVP578: TTGCATGCCTGCAGGTCG, which has an internal SphI site and MVP581: GACTGGCGCGCCTACCTTTGGGTCCTTTGGC, which introduces an AscI site). Then, the *_p_odr-1* fragment was subcloned into the SphI-AscI fragment from *nlg-1::GFP11*^75^

### METHOD DETAILS

#### LTM assay

The odor training protocol contains three 80-minute cycles of training with odor, or a control buffer interspersed with two 30-minute periods of feeding with OP50 *E. coli* bacterial. One day old adult worms were washed with S basal buffer (0.1M NaCI, 0.05M K_3_PO_4_, pH 6.0) off 10 cm NGM plates and into microfuge tubes, where they were washed three times with S basal buffer. The animals were split in two groups and one group was added to a microfuge tube of S basal and the other group was added to a microfuge tube of 1:10,000 dilution of butanone in S basal. The microfuge tubes were then rotated on a rotisserie for 80 minutes.

To make concentrated OP50 for the 30-minute feeding cycles, 100 mL of LB was seeded with single colony of OP50 and was shaken overnight at 37°C at approximately 250 RPM until it reached an OD of approximately 10 and then centrifuged at 4000 RPM for 30 minutes or allowed to settle for 1 hour, the pellet was resuspended in 32 mL of S basal. Animals were washed three times with S basal and then added to a microfuge tube with 750-1000 μL of concentrated OP50 and rotated for 30 minutes. Animals are then washed three times with S basal, allowing the animals to settle five minutes each time, before beginning the next 80-minute cycle of training. After the third treatment cycle, worms were washed three times with S basal and placed on NGM plates with OP50. For single-cycle odor training (Figure 4), the adult worms were washed with S basal from the 10 cm NGM plates and trained for 80 minutes. Further variations of the LTM assay are described in Figure 4.

#### Chemotaxis assay

Chemotaxis plates were made using these materials (for 100 mL of media): 100 mL ddH_2_O with 1.6 g of Difco agar, agar agar, or bacto agar, 500 μL of 1M K_3_PO_4_ solution (pH 6.0), 100 μL 1M CaCl_2_ and 100 μL 1M MgSO_4_. The agar was boiled or autoclaved to dissolve uniformly in 100 mL ddH_2_O and cooled to 57-53°C (to avoid precipitation) before adding the salt solution. 10 mL of media was poured into 10 cm plastic petri dishes and let it cool to solidify. Assay plates were set up as shown in Figure 1A. 1 μL of (1 M) NaN_3_ was pipetted onto the middle of the odor and diluent (control) spots. We then added 1 μL of 200 proof ethanol to the diluent spot. To the odor spot, we added 1 μL of 1:1000 butanone, 1 μL of 1:200 benzaldehyde or 1 μL of 1:1000 diacetyl.

One day old adults grown at 20°C on 10cm NGM plates with OP50 *E. coli* lawns were trained and used for chemotaxis assays. It is critical that the strains be completely clean with no fungal or bacterial contamination. When plating animals after three cycles of training, we did two washes with S Basal buffer and then a third wash with S Basal or ddH_2_O. We plated 50-400 animals at the origin of the plate (bottom) and wicked away excess moisture with a Kim Wipe, being careful not to cause any gouges in the agar, which can cause burrowing. Worms were allowed to roam at least 90 minutes.

To calculate a chemotaxis index (CI), we counted how many worms were in each arena, and how many total worms there were on the plate outside the origin. We subtracted the number of worms in the diluent arena from the number in the odor arena, and then divided that by the total number of worms on the plate that were not at the origin. We censored the assays in which the buffer-trained populations exhibited a CI of less than 0.5, as this indicates that the worms were unable to chemotax to the odor for some reason.

The majority of a buffer-exposed population is attracted to the odor butanone, and their chemotaxis index (CI) is usually between 0.6 and 0.9 while the butanone-exposed population gives rise to a lower, sometimes negative CI between 0.4 and −0.7 (Figure 1B, left). Each point on the graphs represents a CI resulting from an independent day’s population of >50 animals. By subtracting the CI of the buffer-trained population from that of its siblings in the butanone-trained cohort immediately after training, we quantify how much the population has learned (Learning Index, LI). We found that repeated training typically produces LIs from 0.4 to 1.2 (Figure 1B, right, 0 hour after training).

#### Sleep analyses

##### MultiWormTracker and MatLab

The PDMS WorMotel was 3D printed using ProtoCam (PolyJet 3D printing) (https://www.protocam.com/additive-manufacturing-services/polyjet-3d-printing/). In this technique UV light etching is used to produce a glossy finish for finer details. We filled the mould with PDMS to make the motel, We baked it 6 hrs at 70 degree C in baking oven) to maintain elasticity and plasma bonded for 5 minutes. Hydrophobicity stops molten agarose being soaked from the wells, and this also eliminates the need for Tween2 0 to reduce surface tension.

To fill the 48 well WorMotel^55^ (http://fangyenlab.seas.upenn.edu/links.html) with agar, we made 100 mL NGM by adding together 1.8 g low melting-point agarose, 0.3 g NaCl, 0.25 g bacto peptone, and 1μL tween 20 (to keep a flat agar surface). We boiled the media in the microwave, cooled it down to ~50-58°C, then added 100 μL CaCl_2_, 100 μL cholesterol dissolved in EtOH, 100 ul MgSO_4_, and 2.5 mL K_3_PO_4_. We next added 17 μL/well of chip and let it cool. Once the agar was solidified, the worMotel was placed in a transparent Petri dish with 10 mg of gel soil (Soil Moist granules) soaked in 100 mL of water to prevent cracking of wells. Four clay balls were used to prop the lid open uniformly for 1-8 hours or as long as the worms were assayed. Food from NGM/OP50 plate was scooped and smeared on top of the wells to feed worms.

We measured the length of time an individual animal remained quiescent using a frame by frame subtraction method and a frame rate of 3 seconds between frames.^55^ If the pixel displacement was zero from one frame to the next and remained zero for 9 consecutive frames (27 seconds), then a blue line marked this as a quiescent bout on the raster plot. Conversely, when pixel displacement between the consecutive frames was greater than zero, a yellow mark noted 3 seconds of movement (Figure 1C).

Teledyne Dalsa PT-21-04M30 Camera Link Camera (Dalsa Proprietary Sensor 2352x1728 Resolution Area Scan Camera) attached with a Linos Rodagon Modular-Focus lens (f = 60mm) was used to image the entire WorMotel. To obtain a focused working distance, four metal posts with a plastic stage was built. A T175 tissue culture flask filled entirely with water was added to make a cooling chamber as well as a light diffuser (water diffracts light). We used the Multiple-Worm Tracker (MWT 1.3.0r1041) made by Rex Kerr to automate image capture and record worm movement every 3 seconds for the entire duration of the experiment. Irfanview (developed by Irfan Škiljan) was used to re-index the images for sequence verification before quantifying the movements in MatLab. Once indexed using IrfanView, the images were batch processed in MatLab for thresholding each worm uniformly to quantify quiescence using a graphic user interface (GUI) created by MC and CFY and available at https://github.com/LEtoileLab/Sleep_2022.git. After thresholding, the animals were quantified for quiescence and activity using another MatLab code created by RC available at https://github.com/LEtoileLab/Sleep_2022.git

##### WormLab: Speed, Track, Idle Time and Midpoint-Bending Analyses


$$Midpoint\;Bending\;Angle\;(∡\;\Theta_{M})=[180-\{(∡\;\Theta 1)+(∡\;\Theta 2)\}]$$

Using WormLab, we analyzed the WorMotel videos (1 frame per 3 secs). We specifically tagged individual animals belonging to naïve, buffer, butanone and heat-shock animals, and converted them into a 17-point skeleton to measure the bending angle. Therefore, the mid-point bending angle is measured using the 9^th^ point of the skeleton (Figure 1E) as one of the vertices of the triangle where the beginning (1^st^) and the endpoint (17^th^) of the skeleton forms the other two vertices of a triangle.

Using the 9^th^ point as the center of the worm, we measured the trajectories, speed, and track length. To measure the stillness of the worm, we overlayed 10 consecutive frames when an animal started showing the lowest moving average speed, which was cross confirmed using mobility and the changes in mid-point bending angle as shown in the workflow (Figure 1E).

#### WormLab: Posture analysis

Movement quiescence was defined as the 30 second period (10 frames) during which the animal exhibited its lowest moving average speed (e.g., the butanone trained animal in Figure 1E). Images of animals in each 10 second frame were skeletonized and aligned to their midpoints in order to visualize their posture Figure 1E.

#### Arousal latency and body bends after arousal analyses

We assessed animal responsiveness to a stimulus which has been previously shown to disrupt quiescence in *C. elegans* (Nagy et al., 2014). Specifically, 3.5 cm or 5.5 cm diameter NGM plates with OP50 lawns with 20-30 worms were placed on a 50mm piezo 1.2 KHZ piezo buzzer elements (Digikey #668- 1190-ND). Piezo elements were supplied with 5V with a 50% pulse-width modulated duty cycle using an Arduino-style microcontroller and its accompanying software, using the code named “Arduino_blink_buzz” created by RLD accessible at www.GitHub.com/letoilelab/Sleep_2022. Stimulus onset was synchronized with video recording by flashing a blue LED, used at the maximum light intensity (we used Digikey #1528-2334-ND) at a distance of about 10cm during video recording. In cases where animals were exposed to prolonged stimulation, animals were subjected to blocks of stimulation for 5 minutes with the blue light flashing for 1 second every 20 seconds, followed by no stimulation for 5 minutes. Videos were recorded on an Imaging Source DMK 23GP031 camera using Micromanager software.^105^ Videos were scored by four independent investigators who each scored all the videos and the mean of their arousal thresholds and body bend analyses were used.

#### Pharyngeal Pumping Assay

This assay was performed by standard methods.^106^To perform the assay, we watched the pharynx of a worm under a stereomicroscope at 100-200X magnification and once the grinder in the terminal bulb does one complete contraction and relaxation, or “pump”, we counted that as one pump, using a counter to count every time they complete a full pump for 15 seconds. Then, we disposed of the worm to prevent re-counting of the same animal. We took the number of pumps completed and multiplied that by 4 to find the pharyngeal pumping rate in pumps per minute.

To assess the fraction of a population that ceased pumping, we used a similar method to that described by Hill and colleagues in which immobile animals were observed for 1 minute and those that stopped pumping for more than 4 sec were counted as ceasing pumping.^58^

#### Quiescence disruption

Post-training quiescence was disrupted by either mechanical disruption or removal from food for two hours. For mechanical disruption was adapted from Driver and colleagues.^107^ After training the animals were divided into four different groups in addition to performing sleep and chemotaxis assays. The first group was not disturbed for 16 hours, while the second, third and fourth group were mechanically disturbed by plating the worms in a dense bacterial slurry, which was shaken every 15 minutes to ensure uniform movements of the worms. The bacterial slurry was made by centrifuging an overnight culture of OP50 (at 37°C with 250 RPM, OD=10) at 4000 RPM for 15-30 minutes to resuspend the pellet(s) in 5 mL of S Basal. To physically disturb the animals, we shook the plates for one minute out of every 15 (Figure 3Ai). We found that shaking prevented them from sleeping (Video S2). The slurry plates were made by pouring 1 mL of dense bacterial slurry on NGM plates approximately 30 minutes before plating the worms. After mechanically disturbing the worms, the worms were rinsed three times with S Basal before moving them to NGM plates with OP50 bacterial lawns. The third and the fourth group of animals were initially placed on NGM plates with OP50 bacterial lawns for two or four hours, and then washed with S Basal three times before putting them on slurry plates. For removal from food assays, worms were placed on NGM plates without OP50 lawns for the first two hours after training before plating them on NGM plates with OP50 lawns for 14 hours.

To load the WorMotel with worms that did not have food, we placed the worms on NGM plates without food and loaded individual worms into the WorMotel using a pick. This WorMotel was divided into four groups to compare the amount of sleep, where one group of buffer- and butanone-trained animals received food after training and the other group of trained animals did not.

#### Increasing Post Training Sleep

We found that animals that had only one cycle of swimming in butanone showed the lowest mean total quiescence (median of 13.19, Figure 4B, lightest pink, last bar) and those that had three cycles of swimming either in odor or in food showed more quiescence (median of 16.21, 15.94 and 16.01 minutes, Figure 4B red and medium pink, first three bars). Thus, sleep, as measured by total quiescence, is significantly increased if the number of cycles of training is increased from one to three. We then asked if this would convert the short-term into a long-term memory. We found that the cohorts that had two cycles of food training before one cycle of butanone training (third row in 4A) exhibited more long-term memory (LI = 0.73) than one cycle-trained animals. Thus, inducing sleep after a single cycle of odor training was sufficient to increase memory retention after 16 hours.

#### Colony Forming Units Measurement

After the 0-2 hours period of mechanical disturbance with GFP expressing OP50 slurry, worms were washed three times with S Basal before treating 15 minutes with 200 μL 0.5% bleach solution in a 96-well plate. After 15 minutes of incubation at room temperature (20°C), the worms were washed three times with S Basal. The alimentary canal of the worms was dissected out using two 22-gauge hypodermic needles under the dissecting microscope and transferred to the other wells containing 200 μL SOC media, which was incubated at 37°C for 15 minutes before plating the 200 μL of SOC media on LB plates. As a negative control, worms were treated with a bleach solution but left undissected, and treated with SOC media like the dissected worms, which were then plated to confirm that there were no green colonies.

#### Heat Shock Assays

*C. elegans* animals were heat-shocked at 37°C for 5 minutes in a water bath while on 5.5cm unseeded NGM plates covered in Parafilm. After the heat shock, animals were put at 20°C until behavior was assessed by the chemotaxis assay.

#### Calcium imaging

Calcium imaging of the AWC^ON^ neuron was performed on lines expressing the genetically-encoded calcium indicator GcaMP3^70^ under the *str-2* promoter (*JZ1795/pyIs701(_p_str-2::GcaMP3; _p_ofm-1::GFP; _p_ceh-36::mCherry)*). One-day-old adult worms were conditioned to either buffer or 1.23mM butanone diluted in S Basal buffer (the same concentration as used for the butanone conditioning mentioned in “chemotaxis assay”) during three, 80-minute training cycles (interspersed) with feeding (described in “LTM chemotaxis assay”). Immediately after the end of the third training cycle or after a 16-hour overnight period on food, worms were rinsed three times in S Basal buffer and loaded into a custom, polymer polydimethylsiloxane (PDMS) microfluidic device.^108^ The nose of the animal was exposed to liquid streams of either S Basal buffer or 1.23mM butanone. A manual switch attached to a solenoid valve was used to direct the buffer or odor stream across the nose of the worm. The stimulation protocol consisted of exposing worms to S Basal buffer for 30 seconds followed by a 30-second exposure to 1.23mM butanone (odor on) or by exposing worms to 1.23mM butanone for 30 seconds followed by a 30 second exposure to S Basal buffer (odor off). Fluorescence was monitored with a Zeiss 40X air objective on an inverted microscope (Zeiss Axiovert 200). Images were taken at 2 frames per second with a blue light exposure time of 100ms using an ORCA-Flash 2.8 camera (Hamamatsu).

#### Calcium imaging analysis

Fiji software was used with the Multi Measure plugin to analyze the images. In animals expressing the GcaMP3 reporter in the AWC^ON^ neuron (*JZ1795/pyIs701(_p_str-2::GcaMP3; _p_ofm-1::GFP; _p_ceh-36::mCherry)*), the ROI was established at the center of the AWC cell body. A background ROI was also taken, just outside of the animals. Then, the mean fluorescence intensity at the background ROI was subtracted from the mean fluorescence intensity at the cell body ROI and that serves as the “F” values. The fluorescence intensity of the GcaMP3 reporter in the first three images is defined as F_0_. ΔF is the F_0_ value subtracted from each F value. For every worm imaged, the mean of the ΔF_0_/F (%) values is taken from 10 seconds before and after the butanone is turned on or off (i.e., the means at 20-30 seconds and 30.5-40.5 seconds are taken). The delta is then taken between the two means and the absolute value is taken of that number for comparisons between the datasets (e.g., buffer vs butanone-trained cohorts) taken on the same day.

#### Generating the GRASP Imaging Strain

To visualize AWC-AIY synapses, we generated a construct driving expression of NLG-1::GFP11 in AWC neurons and coinjected it with a construct driving expression of NLG-1::GFP1-10 in AIY neurons. An additional construct drove expression of cytosolic dsREDII in the AWC neurons^102^ to visualize AWC neurites. We generated transgenic animals carrying these markers, integrated the marker into the genome, and outcrossed background mutations. AWC neurons have dendrites that extend to the nose of the worm, and axons that extend into the nerve ring, which forms an arc in the head of the worm [Figure 6B; White et al.^15^].

#### LTM NLG-1 Synaptic Imaging Assays

The LTM training paradigm described above was modified to accommodate NLG-1 GRASP imaging. Approximately 30 NGM plates of day one gravid adult *iyIs35* worms were prepared to allow enough worms for multiple batches and imaging. Worms were divided into four batches that began training 40 minutes apart. Batches one and three were trained in a control buffer (S Basal), while batches two and four were trained in the odor solution. For all LTM NLG-1 GRASP synaptic imaging assays, images of animals were only analyzed for NLG-1 GRASP intensity from training batches that passed the behavioral batch chemotaxis tests. Specifically, butanone-trained batches allowed to sleep were considered to pass the behavioral test if they were not attracted to butanone (CI<0.5), while butanone-trained batches with disrupted sleep and buffer-trained batches were considered to pass the behavioral test if they were attracted to butanone (CI>0.5).

For all NLG-1 GRASP LTM assays, A Zeiss Axio Imager.A1 compound fluorescent microscope (Figures 6F, 6G, 7B, S5C, S6A, S6B, and S7D) and a Zeiss LSM710 confocal microscope (Figures 6C, 6D, 6E, 7A, S7A, and S7B) were used to capture images of live *C. elegans* under 630X magnification. For batch assays (all assays except the single-worm time course assays), images were taken of approximately 20 animals from each batch within approximately 20 minutes of the time point.

LTM NLG-1 GRASP 16-hour mechanical disturbance assays: After three cycles of training (described above), half the worms from each batch were placed on NGM plates with an OP50 *E. coli* lawn. The other half were placed on plates with 1 mL of OP50 suspended in S Basal buffer (as described above) and tapped for one minute out of every 15 minutes for a two-hour period. Worms on these plates were then transferred to NGM plates with OP50 lawns after the two-hour period. All plates were incubated at 20°C until 16 hours after training, when worms were washed (as described above). 50-400 worms from each of the eight batches underwent the butanone chemotaxis assay. Worms were anesthetized for imaging on 2% agarose pads using a 2:1 ratio of 0.3 M 2,3-butanedione monoxime (BDM) and 10 mM levamisolein M9 buffer. All micrographs taken were of one-day old and two-day old gravid adults.

LTM NLG-1 GRASP 16-hour removal from food assays: After three cycles of training (described above), half the worms from each batch were placed on NGM plates with an OP50 *E. coli* lawn, and half were placed NGM plates without bacteria, then transferred to NGM plates with an OP50 E. coli lawn after two hours. All plates were incubated at 20°C until 16 hours after training, when worms were washed (as described above). Approximately twenty worms from each of the eight batches were anesthetized and imaged using a Zeiss Axio Imager.A1 compound fluorescent microscope and Axiovision software, as described above, and 50-400 worms from each of the eight batches underwent the butanone chemotaxis assay.

LTM NLG-1 GRASP batch 0-hour, 2-hour, and 16-hour assays: After three cycles of training, worms from batches one to four were each divided into three groups so that imaging and chemotaxis experiments could be performed at three timepoints: zero hours after training, two hours after training, and 16 hours after training. 0-hour Imaging and Chemotaxis: After training, worms from all four batches were washed (as described above). From each batch, ~20 worms were separated, anesthetized, and imaged under the Zeiss Axio Imager.A1 compound fluorescent microscope and Axiovision software, synapse imaging analysis as described above, and 50 to 400 worms were assessed for butanone chemotaxis. Two-hour Imaging and Chemotaxis: After training, worms from batches one and two were each placed on NGM plates with an OP50 lawn. After two hours, animals were washed as described above. Approximately twenty animals from each of these batches were anesthetized and imaged, and 50 to 400 worms from each of these batches were assessed for butanone chemotaxis. 16-Hour Imaging and Chemotaxis: After training, worms from batches one, two, three, and four were each placed on NGM plates with OP50 lawns. After 16 hours, animals were washed as described above. Approximately twenty animals from each of these batches were anesthetized and imaged, and 50 to 400 worms from each of these batches were assessed for butanone chemotaxis.

LTM Single worm time course NLG-1 GRASP assays: The training paradigm detailed in the LTM NLG-1 GRASP assays were repeated as described above with the following modifications following the three cycles of training. All worms from each batch were placed on NGM plates with OP50 lawns and gravid adults with only one row of eggs were selected for the single worm time course assay. Each animal was either imaged at 0 and 2 hours after training, or at 2 and 16 hours after training. Animals were anesthetized with 10mM tetramisole in a 1:1 ratio with M9 buffer or a 2:1 ratio of 0.3 M 2,3-butanedione monoxime (BDM) to 10mM tetramisole on agarose pads or NemaGel. After imaging at the first timepoint, each animal was picked from the agarose pad into an approximately 50 μL drop of M9 buffer on an NGM plate with an OP50 lawn. The animal was then transferred to a drop of M9 buffer on a second NGM plate with an OP50 lawn to aid the recovery from the anesthetic. The worms spent roughly 30 minutes total in M9 buffer. These plates were incubated at 20°C until the second timepoint.

For animals imaged at 0 and 2 hours after training, animals were again anesthetized and imaged as described above for the first time point. For animals imaged at 2 and 16 hours after training, at the 16-hour timepoint, each animal that was mobile (as demonstrated by movement tracks on the bacterial lawn) was tested for chemotaxis to butanone before imaging. Individual buffer-trained animals that were attracted to the odor, and butanone-trained animals that were not attracted to the odor were then imaged again as described above. Single animals were washed for five minutes each in a drop of S Basal buffer and a drop of ddH_2_O. Single animals were then placed on a single worm chemotaxis plate with the origin at the center of the plate. The single worm chemotaxis plates were made as previously described (see “chemotaxis assay” section above) but with the origin of the single worm placement in the center of the plate. The worm was allowed to roam on the plate for at least 10 minutes. For animals to pass this single-worm behavioral screen, buffer-trained worms needed to move directly towards butanone or stay on the butanone side of the plate the majority of the time, while butanone-trained worms needed to move and not make directed movement towards butanone nor spend the majority of time on the butanone side of the plate. Only animals from assays with two or more successful buffer- and butanone-trained animals that passed the single-worm behavioral screen at the 16-hour timepoint were included. Additionally, only micrographs of animals with their head approximately on its side at both timepoints were analyzed.

#### Synapse imaging analysis

NIH ImageJ software^109^ was used to analyze all Zeiss Axio Imager.A1 compound fluorescent micrographs taken for AWC-AIY NLG-1 GRASP phenotypic quantification, as previously described.^75,76^ In brief, AWC-AIY NLG-1 GRASP intensity was quantified by measuring^93^ fluorescence intensity through circling punctal clusters. In Figures 6F, 6G, 7B, S5C, and S6B median intensity values for each treatment were normalized to fluorescence intensity levels for buffer-trained animals placed on food for 16 hours on the same day. In Figure S7D, for the single-worm assays, the median intensity of each worm at the first timepoint was normalized to 100% and the difference in intensity between the first and second timepoint was quantified for each animal. Since animals that are imaged twice can undergo photobleaching, we assessed the proportion of animals with greater than or equal to 50% reduction in fluorescence intensity between the two timepoints.

PHB-AVA NLG-1 GRASP intensity in Figure S6D was also measured by outlining clusters of puncta. Background fluorescence intensity near the gut was also taken into account by calculating the minimum intensity value in the area directly surrounding the puncta. This background intensity value was subtracted from the intensity for each pixel in the punctal cluster, and the adjusted values were added as previously described.^75,76^

All team members performing image analysis were blind to the animals’ prior conditioning. Animals that appeared unhealthy or that moved too much during image acquisition were not analyzed.

### QUANTIFICATION AND STATISTICAL ANALYSIS

For Figures 1, 2, 3, 4, 5, and S1-S4, statistics were performed using GraphPad Prism 8 and Rstudio. *P*-values are used for the statistical readouts, with the following notations: NS P>0.05, * P<0.05, ** P<0.01, *** P<0.001, and **** P<0.0001. All data included in the same graph were analyzed for type of data distribution with the Shapiro-Wilk normality test. If datasets were normally distributed, then one-way ANOVA was used for multiple comparisons, followed by Bonferroni’s multiple correction. If the data were found to be non-normal, then the non-parametric Kruskal-Wallis test was used to analyze differences in means, followed by pairwise comparisons using the Mann-Whitney two-tailed U-test. Then, to correct for Type I error, the Hochberg test was run on p-values compared in the same graph to adjust the p-values for multiple comparisons, which often conservatively increases p-values to avoid incorrectly rejecting the null hypothesis. For graphs with only two datasets to compare, the Shapiro-Wilk test was performed, followed by the t-test or U-test, depending on the distribution of the datasets. For correlation data, Pearson’s correlation test was used. The standard error of the mean (SEM) was calculated and shown on each graph. Specific statistical tests used for each graph in the manuscript are included in the figure legends. Additional statistical analysis can be found in Table S1.

For Figures 6, 7, and S5-S7, statistics were performed using R and Excel. *P*-values are similarly used for the statistical readouts. For normally distributed data, one-way ANOVA was used to analyze variance between treatments. If the p-value was less than 0.05, then pair-wise t-tests were used to compare the means of each pair of groups. For nonparametric data, the Kruskal-Wallis test was used to analyze variance between treatments. If the p-value was less than 0.05, then the Mann-Whitney U-test was used to compare the medians of each pair of groups. If more than one t-test or U-test was conducted, the resulting *p*-values were adjusted for multiple comparisons using the Hochberg method, which is standardly applied to adjust for the tendency to incorrectly reject a null hypothesis when multiple comparisons are made, and can only conservatively increase *p*-values.

To test if the rate of synaptic change was significantly different between 0-2 hours and 2-16 hours after training for butanone-trained and buffer-trained animals (separately), we fitted a continuous, piece-wise-linear spline least squares regression model to the square root transformed response separately for butanone- and buffer-trained animals as a function of time. The square root transformation was necessary to satisfy the model assumption of normally distributed residuals. For this spline function, one can test whether the slope between 0-2 hours and the slope between 2-16 hours is significantly different. In both cases the slope differs significantly (p-value is 0.00410 for buffer-trained and 0.00175 for butanone-trained animals). This indicates that the rate of synaptic change in the first two hours after training is significantly different than the rate of synaptic change in the following 14 hours.

For single worm LTM NLG-1 GRASP statistical analysis in Figure S7D, we performed two-independent sample Z-tests.

Additional statistical details of experiments can be found in figure legends.

## Supplementary Material

MMC7

MMC6

MMC5

MMC4

MMC3

MMC2

MMC1

FigS4

FigS3

FigS2

FigS1

FigS5

FigS6

FigS7

15

## Figures and Tables

**Table:** KEY RESOURCES TABLE

REAGENT or RESOURCE	SOURCE	IDENTIFIER
Bacterial and virus strains		
OP50 *E. coli*	Caenorhabditis Genetics Center	OP50
OP50-GFP *E. coli*	Caenorhabditis Genetics Center	OP50-GFP
Chemicals, peptides, and recombinant proteins		
2-butanone	Sigma-Aldrich	360473
Sodium azide 99%	Fisher Scientific, Sigma-Aldrich	ICN10289180, S2002
Benzaldehyde	Sigma-Aldrich	B1334
Diacetyl/2,3-butanedione	Sigma-Aldrich	B85307
Levamisole	Acros Organics	AC187870100
Nemagel	InVivo Biosystems	Discontinued
BDM	Fluka Analytical	31550/2003485
Tween 20 detergent	Millipore	655204
NaCl	Fisher Chemical	S671-10
Potassium phosphate dibasic	Fisher Scientific	S375-500
Potassium phosphate monobasic	Sigma-Aldrich	P285
Bacto agar	Difco	90000-762
Calcium chloride	Sigma-Aldrich	C8106
Magnesium sulfate	Sigma-Aldrich	M7506
Low melting point agarose	Apex Chemicals and Reagents/Genesee	20-104
Bacto peptone	Difco	DF0118-07-2
Cholesterol	Sigma-Aldrich	C3045
PDMS/ Dow Corning Sylgard 184 Silicone Encapsulant	Ellsworth Adhesives	4019862
Soil Moist granules	JRM Chemical Inc.	N/A
95% Ethanol	Fisher Scientific	A405P-4
Agarose	Fisher Scientific	BP1356-500
Experimental models: Organisms/strains		
wild-type Bristol N2 var.	Caenorhabditis Genetics Center (CGC)	N2
*peIs578 (pnpr-9::casp1; punc-122::mCherry; pnpr-9::venus)*	Iino lab	AIB kill; strain name: JN578
*ttx-3(ks5) X; peIs578 (pnpr-9::casp1; punc-122::mCherry; pnpr-9::venus)*	This study	AIB, AIY double kill; strain name: JZ2008
*crh-1(tz2) III*	CGC	*crh-1/CREB*; strain name: YT17
*ceh-17(np1)*	CGC	*IB16*
*acy-1(ce2)*	CGC	*KG518*
*wyIs155 (pgpa-6::nlg-1::GFP1-10; pflp-18::nlg-1::GFP11; p^nl-1^::mCherry; pflp-18::mCherry; podr- 1::DsRedII) X*	This study	PHB-AVA NLG-1 GRASP; strain name: MKV1058
*iyIs35 (pttx-3::nlg-1::GFP1-10; podr-1::nlg-1::GFP11; p^odr-1^::DsRedII; punc-122::RFP) III*	This study	AWC-AIY NLG-1 GRASP; strain name: MKV1022
*pyIs701(pstr-2::GCaMP3; pofm-1::GFP; pceh-36::mCherry)*	This study	*pAWCON::GCaMP3*; strain name: JZ1795
Oligonucleotides		
MVP578: TTGCATGCCTGCAGGTCG	This study	Forward primer used to generate *podr-1::nlg-1::GFP11*
MVP581: GACTGGCGCGCCTACCTTTGGGTCCTTTGGC	This study	Reverse primer used to generate *podr-1::nlg-1::GFP11*
Recombinant DNA		
*pttx-3::nlg-1::GFP1-10*	Feinberg et al.^49^	Used to generate *iyIs35*
*podr-1::nlg-1::GFP11*	This study	Used to generate *iyIs35*
*podr-1::DsRedII*	L’Etoile and Bargmann^102^	Used to generate *iyIs35*
*punc-122::RFP*	Loria et al.^103^	Used to generate *iyIs35* and *wyIs155*
*pgpa-6::nlg-1::GFP1-10*	Park et al.^75^	Used to generate *wyIs155*
*pflp-18::nlg-1::GFP11*	Park et al.^75^	Used to generate *wyIs155*
*pnlp-1::mCherry*	Park et al.^75^	Used to generate *wyIs155*
*pflp-18::mCherry*	Park et al.^75^	Used to generate *wyIs155*
Software and algorithms		
Prism 8	Graphpad	https://www.graphpad.com/scientificsoftware/prism/
ImageJ	NIH	https://imagej.nih.gov/ij/download.html
Fiji	Fiji contributors	https://imagej.net/Fiji
RStudio	RStudio	https://www.rstudio.com/products/rstudio/#Desktop
Axiovision	Zeiss	https://www.zeiss.com/microscopy/us/products/microscope-software/axiovision.html
Multi-Worm Tracker	Rex Kerr	https://sourceforge.net/projects/mwt/
Matlab	MathWorks	https://www.mathworks.com/products/matlab.html
μmanager	Ron Vale lab	https://micro-manager.org/wiki/Download%20Micro-Manager_Latest%20Release
Irfanview	Irfan Skiljan	https://download.cnet.com/IrfanView/
ARDUINO 1.8.9	Arduino	https://www.arduino.cc/en/Main/Software
Arduino_code1_blink_buzz-RLD and Arduino_code2_blink_buzz-RLD	This study	www.GitHub.com/letoilelab/Sleep_2022
Sleep analyses_allMatlabCodes	This study	http://www.GitHub.com/letoilelab/Sleep_2022
MC-Quiescence_V1202_GUI_fromCFY	Churgin et al., 2017^55^	http://www.GitHub.com/letoilelab/Sleep_2022
WormLab	MBF Bioscience	https://www.mbfbioscience.com/product-resources/wormlab-resources
Other		
PDMS WorMotel	Churgin et al.^55^	48-well custom PDMS device
PDMS WorMotel	This study	120-well custom PDMS device https://ucsf.box.com/s/471bf85tjl6voq90ihit65lj9zoh6m

## Highlights

- Repeated, spaced odor training induces sleep in *Caenorhabditis* elegans
- Sleep promoted by the ALA neuron is necessary for long-lasting memory
- Memory consolidation requires AIY interneurons, which function in odor seeking
- Sleep and training are required to reduce AWC olfactory neuron synapses with AlYs

## INCLUSION AND DIVERSITY

We support diverse, inclusive, and equitable conduct of research. One or more of the authors of this paper self-identifies as an underrepresented ethnic minority in science. One or more authors self-identifies as a member of the LGBTQ+ community. One or more authors on this paper received support from a program designed to increase minority representation in science.
