# Supplementary figures and images for: Sleep is required to consolidate odor memory and remodel olfactory synapses
Source: Cell. Author manuscript; Available in PMC 2023 Jul 19. (PMC10354834; doi:10.1016/j.cell.2023.05.006)

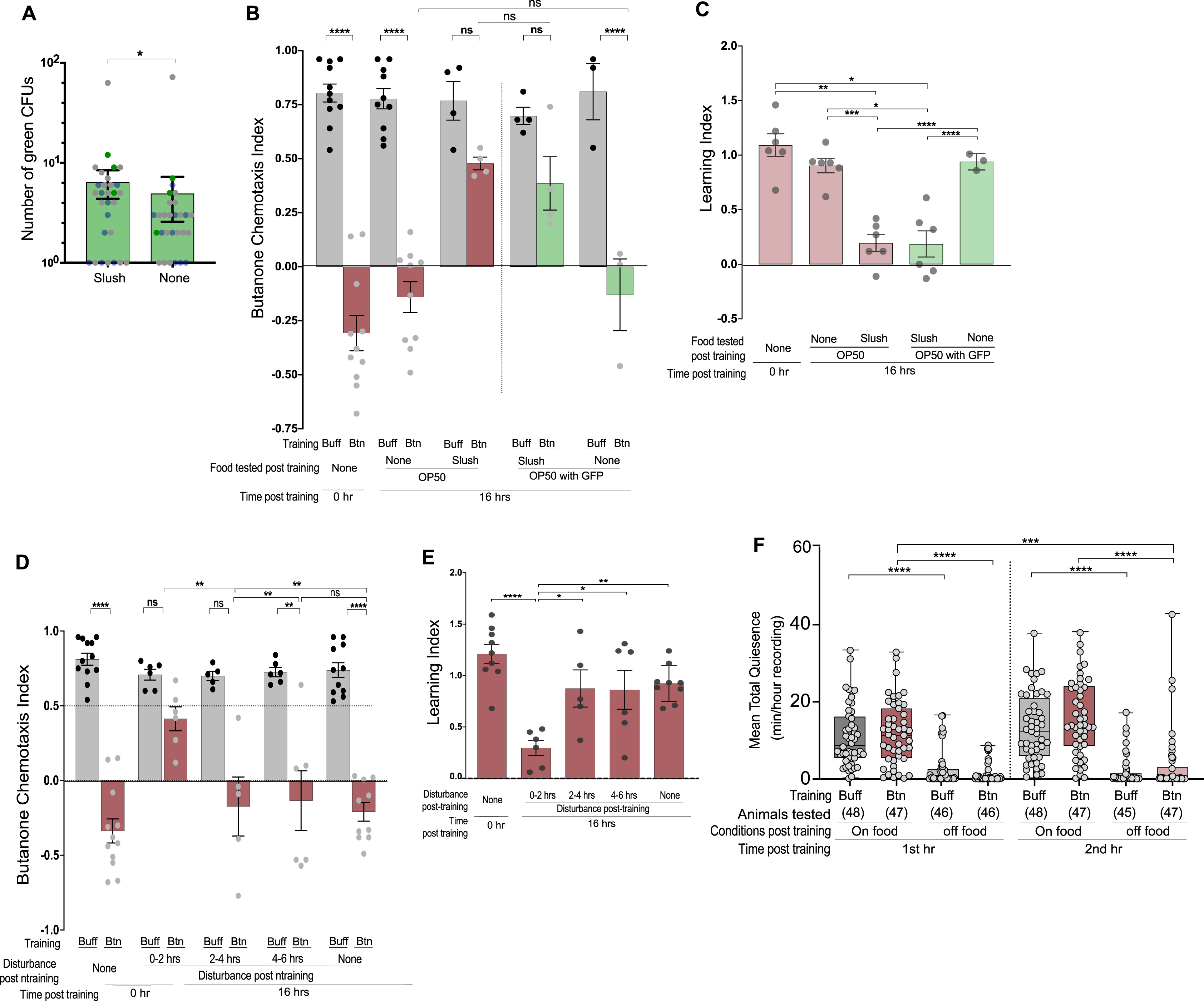

Supplement: FigS4 [file NIHMS1901363-supplement-FigS4.jpg]

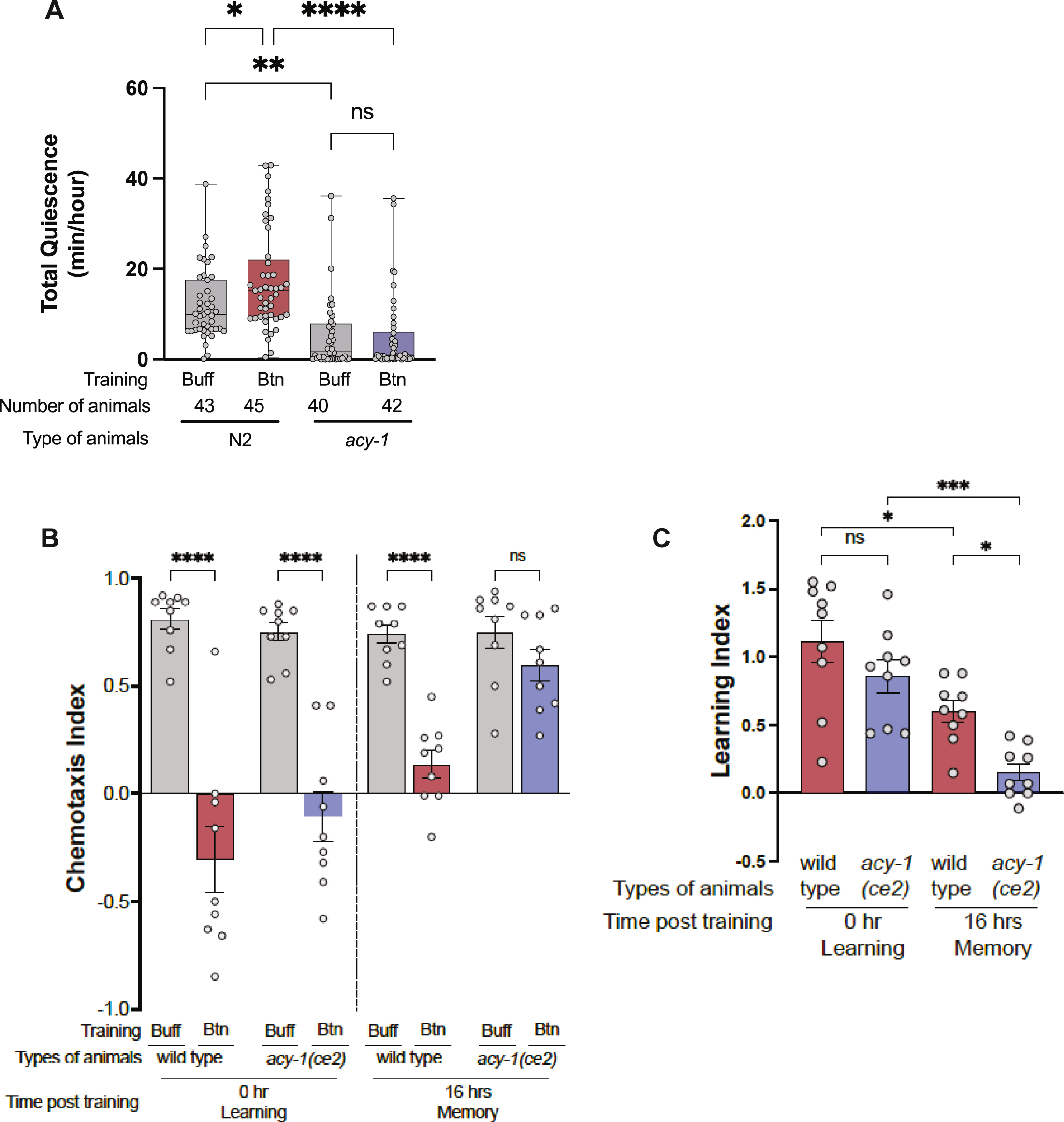

Supplement: FigS3 [file NIHMS1901363-supplement-FigS3.jpg]

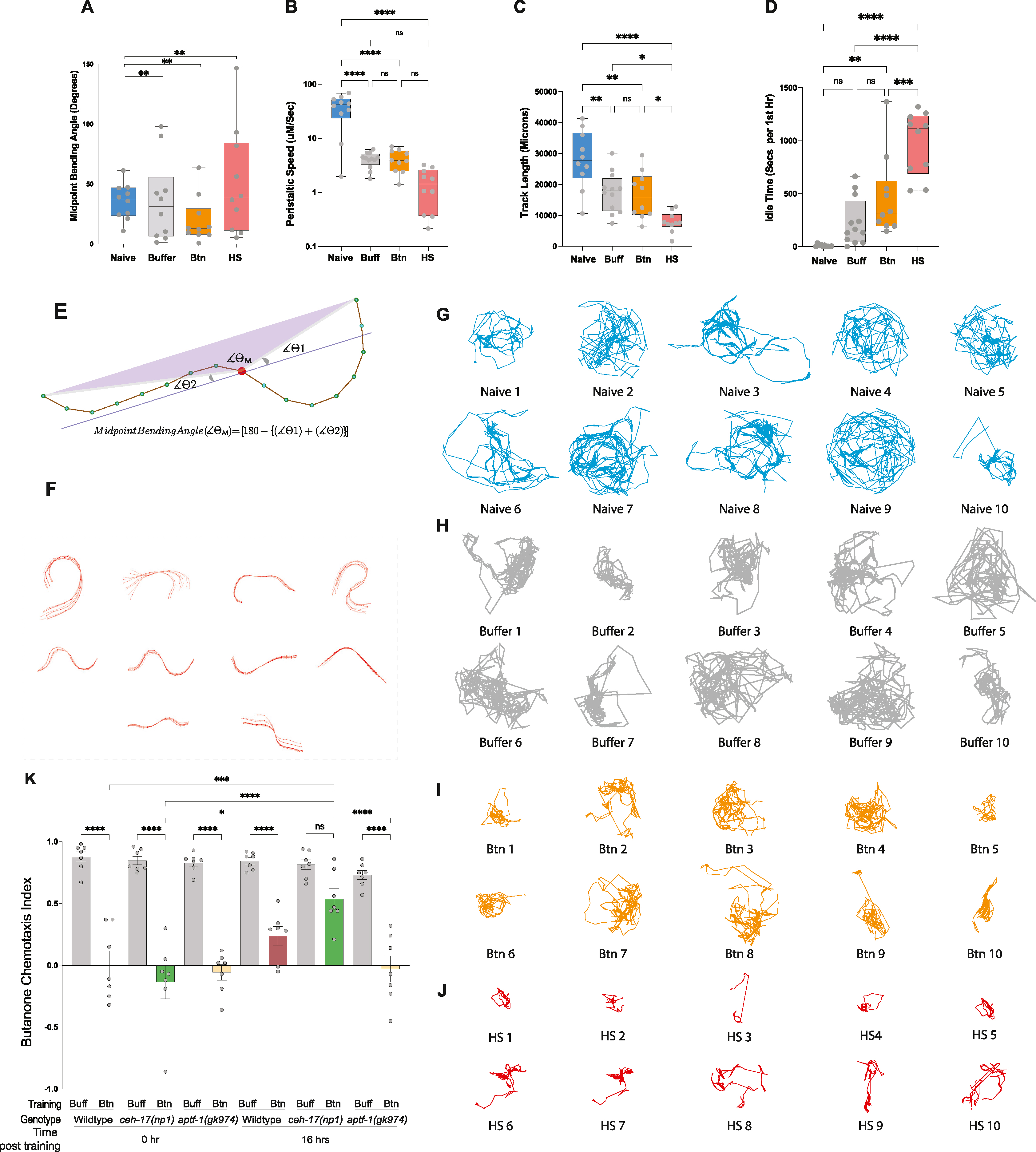

Supplement: FigS2 [file NIHMS1901363-supplement-FigS2.jpg]

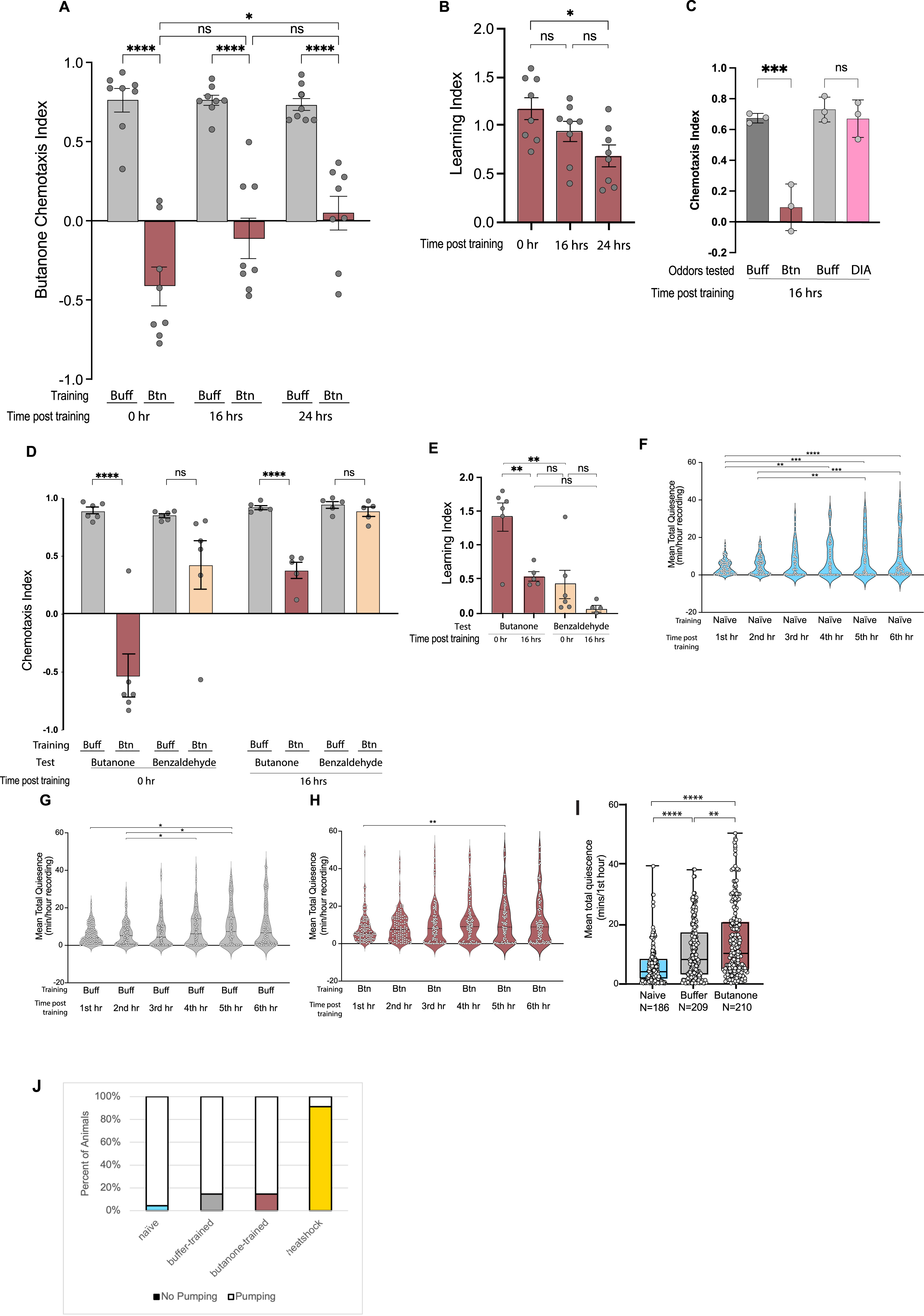

Supplement: FigS1 [file NIHMS1901363-supplement-FigS1.jpg]

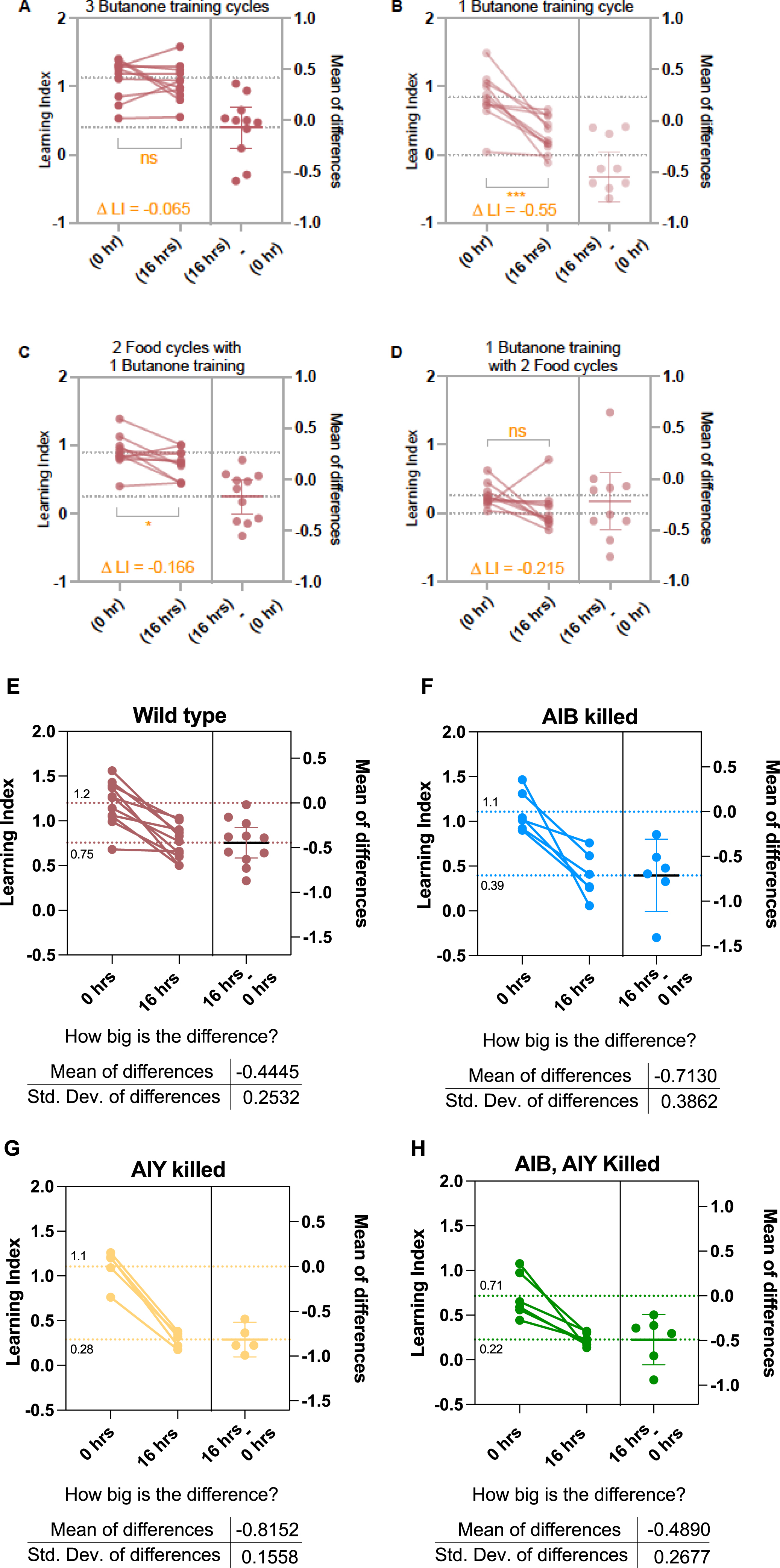

Supplement: FigS5 [file NIHMS1901363-supplement-FigS5.jpg]

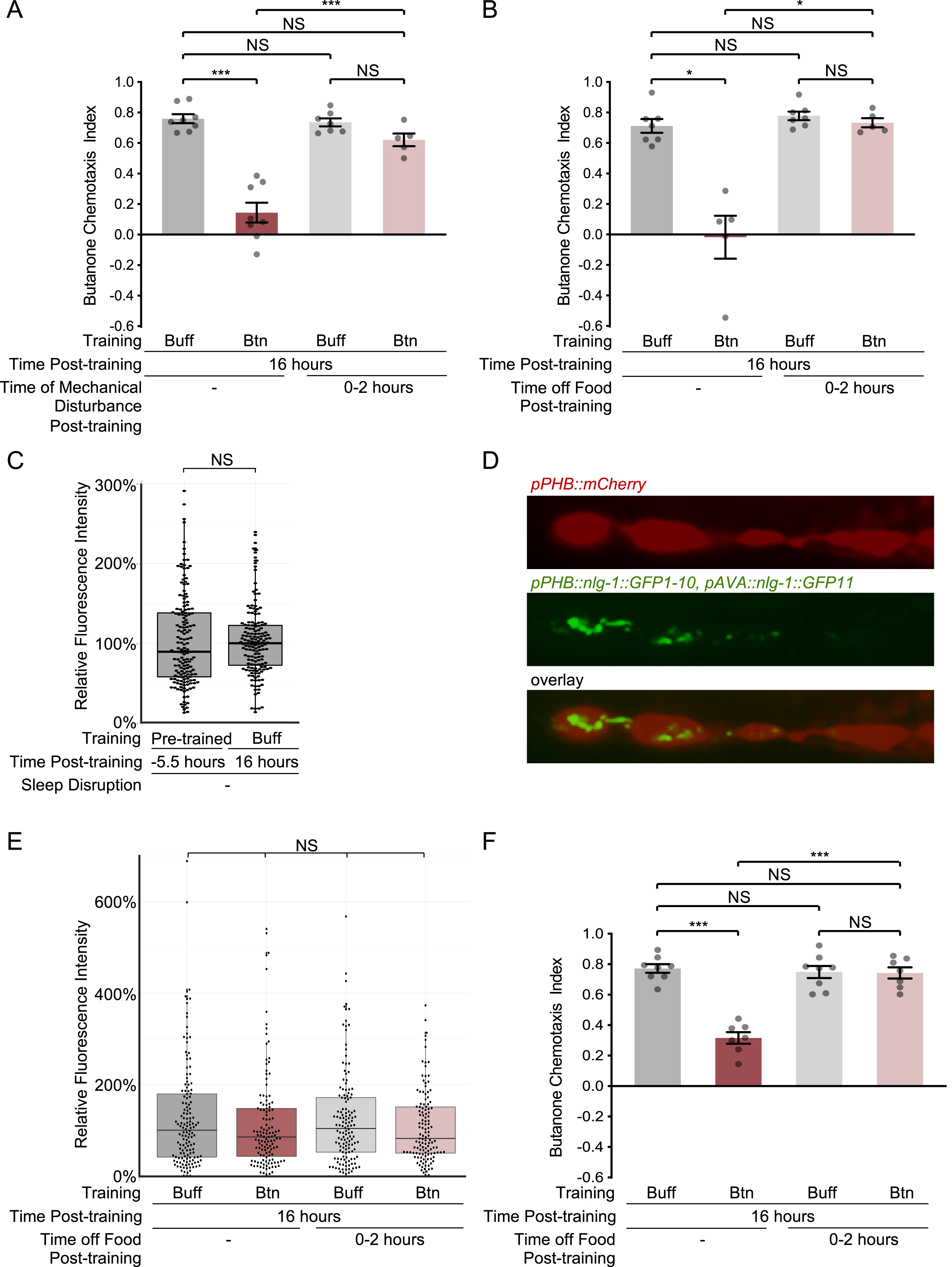

Supplement: FigS6 [file NIHMS1901363-supplement-FigS6.jpg]

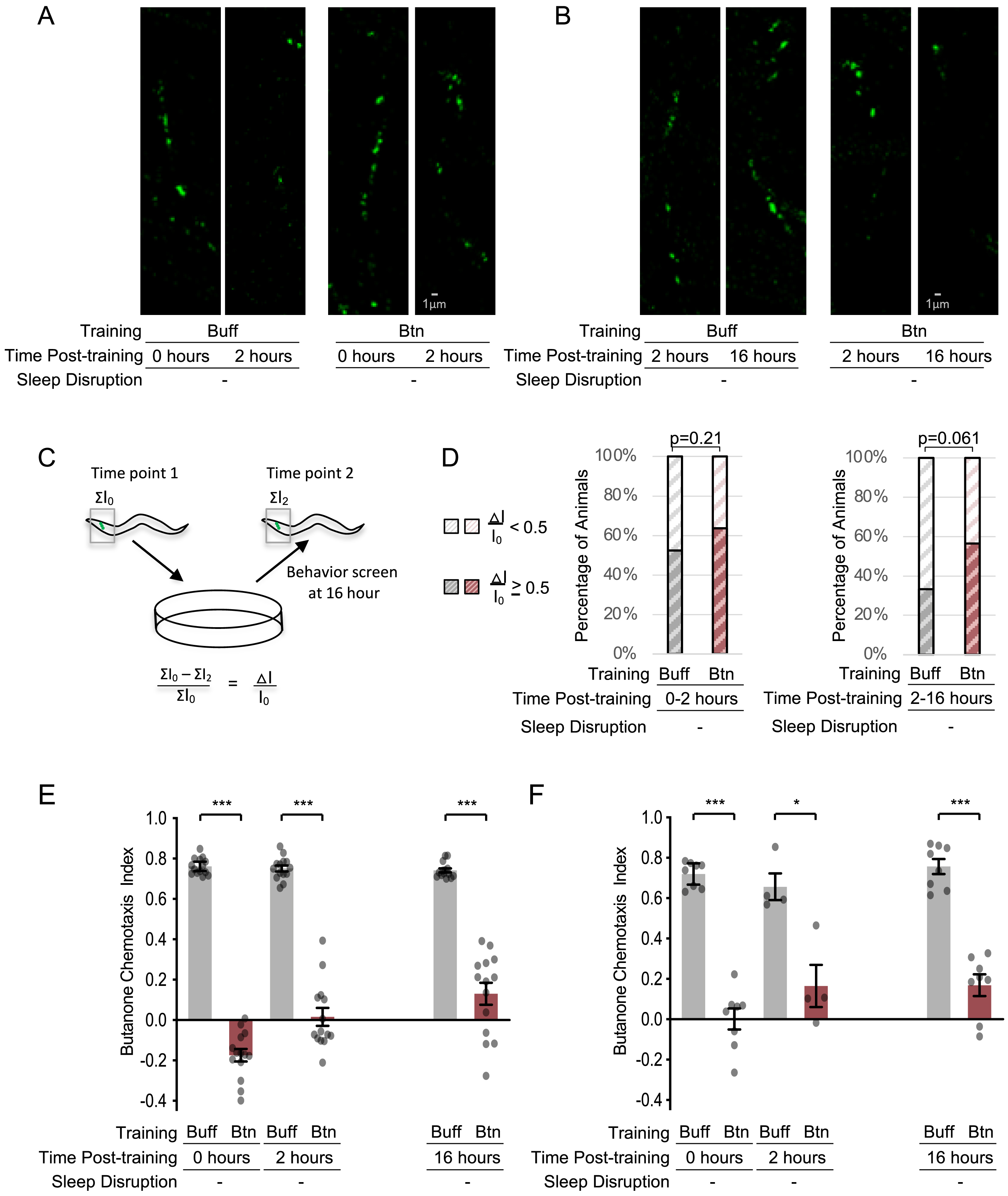

Supplement: FigS7 [file NIHMS1901363-supplement-FigS7.jpg]
