# Supplementary material for: Sleep is required to consolidate odor memory and remodel olfactory synapses
Source: Cell. Author manuscript; Available in PMC 2023 Jul 19. (PMC10354834; doi:10.1016/j.cell.2023.05.006)
Supplement: 15 [file NIHMS1901363-supplement-15.pdf]

CellPress
OPEN ACCESS

Supplemental figures

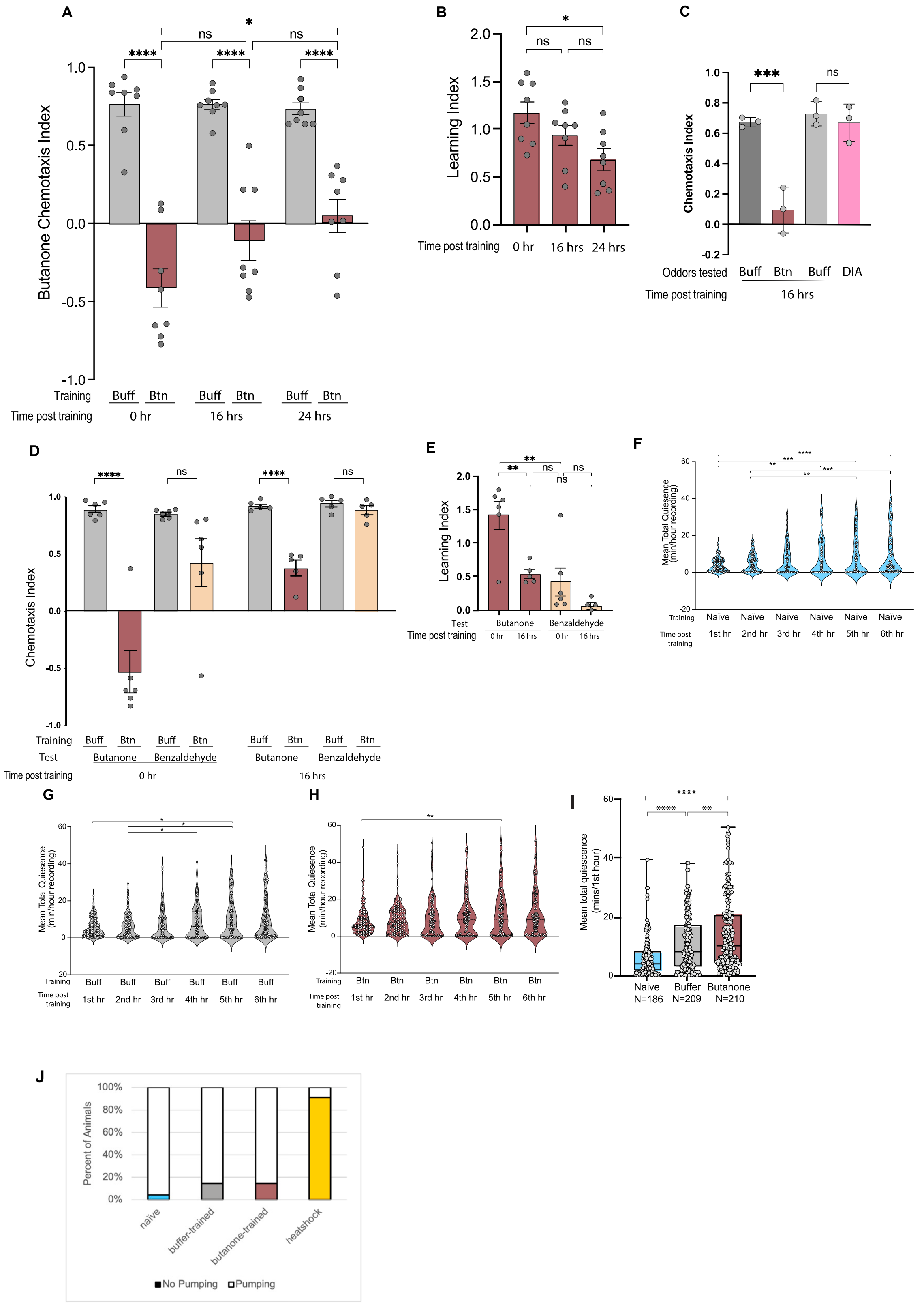

(legend on next page)

Figure S1. Butanone training induces both butanone-specific memory that lasts at least 24 h and movement and feeding quiescence in the first hour after training, related to Figure 1

(A and B) (A) The CIs and (B) the LIs of buffer and butanone trained animals show that memory persists up to 24 h. One-way ANOVA was performed to compare LIs wherein the p values are reported as ****$p < 0.0001$, ***$p < 0.001$, **$p < 0.01$, *$p < 0.05$, and (ns) is $p > 0.05$. N > 7 trials.

(C) At 16 h, butanone-trained animals do not avoid diacetyl. One-way ANOVA with Bonferroni's correction, N = 3 trials.

(D and E) (D) CIs and (E) LIs of three-cycle butanone training does not affect attraction to benzaldehyde after 16 h (showing specificity of butanone sensing). CIs of animals trained with buffer or butanone are tested for attraction to butanone or benzaldehyde 0 and 16 h after training. One-way ANOVA with Bonferroni's correction, p values are reported as ****$p < 0.0001$, ***$p < 0.001$, ** < 0.01, *$p < 0.05$, and (ns) is $p > 0.05$. N = 5 trials.

(F–H) Prolonged stays in a WorMotel induces sleep. However, the butanone-trained animals show the least increase in the amount of quiescence over the course of 6 h in WorMotel because they are already quiescent immediately after training. One-way ANOVA was performed, and p values are reported as ****$p < 0.0001$, ***$p < 0.001$, **$p < 0.01$, *$p < 0.05$, and (ns) is $p > 0.05$. Each gray dot represents the number of animals. N = 7 trials.

(I) The mean total quiescence of animals during the first hour after training. N = 30 trials. One-way ANOVA with Bonferroni's correction, ****$p < 0.0001$, ***$p < 0.001$, *$p < 0.01$.

(J) With heat-shocked animals as a positive control, a recovery plate after training contains two kinds of animals pumping (white portion) and no pumping (colored portions). Percent of animals not pumping are significantly higher than naive. Z test with Hochberg correction (**$p < 0.005$).

CellPress
OPEN ACCESS

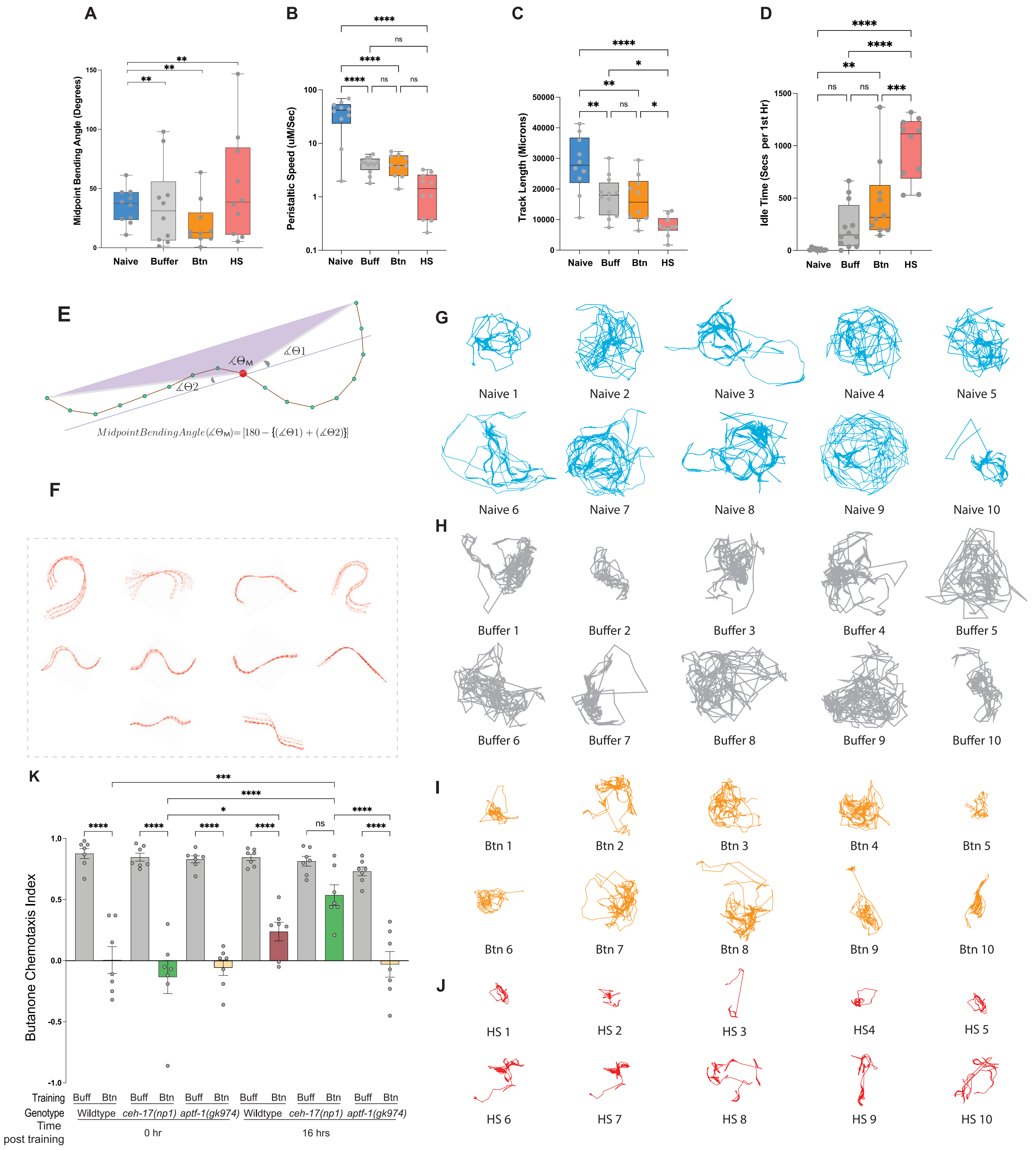

Figure S2. Butanone training induces both reduction in movement and changes in posture that resemble sleep and evokes memory that depend on the ALA but not RIS sleep-promoting neurons, related to Figure 1

(A) With heat-shocked animals as a positive control, the midpoint bending angle of the trained animals is significantly lower than the untrained animals. $^{**}p < 0.005$, one sample t and Wilcoxon signed rank test, N =10 animals.

(B) The equation to measure mid-point bending angle.

(C) Posture found in heat-shocked animals.

(D) The peristaltic speed of the trained animals is significantly lower than the untrained animals and similar to heat-shocked animals. $^{****}p < 0.0001$, one-way ANOVA with Bonferroni's multiple correction, N = 10 animals.

(legend continued on next page)

(E) The track length of the heat-shocked animals is lowest, and the track length of the trained animals is significantly lower than the untrained animals. ****$p < 0.0001$, one-way ANOVA with Bonferroni's multiple correction, N = 10 animals.

(F) The idle time is an independent confirmation of the WormLab software and the MATLAB script of the WorMotel showing that butanone-trained populations sleep more than naive, and the heat-shocked animals being a positive control for sleep, are most quiescent. ****$p < 0.001$, one-way ANOVA with Bonferroni's multiple correction, N = 10 animals.

(G–J) The track trajectories during first hour post-training of naive, buffer, butanone and heat-shocked animals are shown. The trajectories become small as each animal starts sleeping more.

(K) The CIs of wild type, *ceh-17(np1)*, and *aptf-1(gk974)* are shown. Two-way ANOVA with Bonferroni's multiple correction, N = 7 trials. p values of the mean total quiescence of animals during first hour after training are reported as ****$p < 0.0001$, ***$p < 0.001$, **$p < 0.01$, *$p < 0.05$, and (ns) is $p > 0.05$.

CellPress
OPEN ACCESS

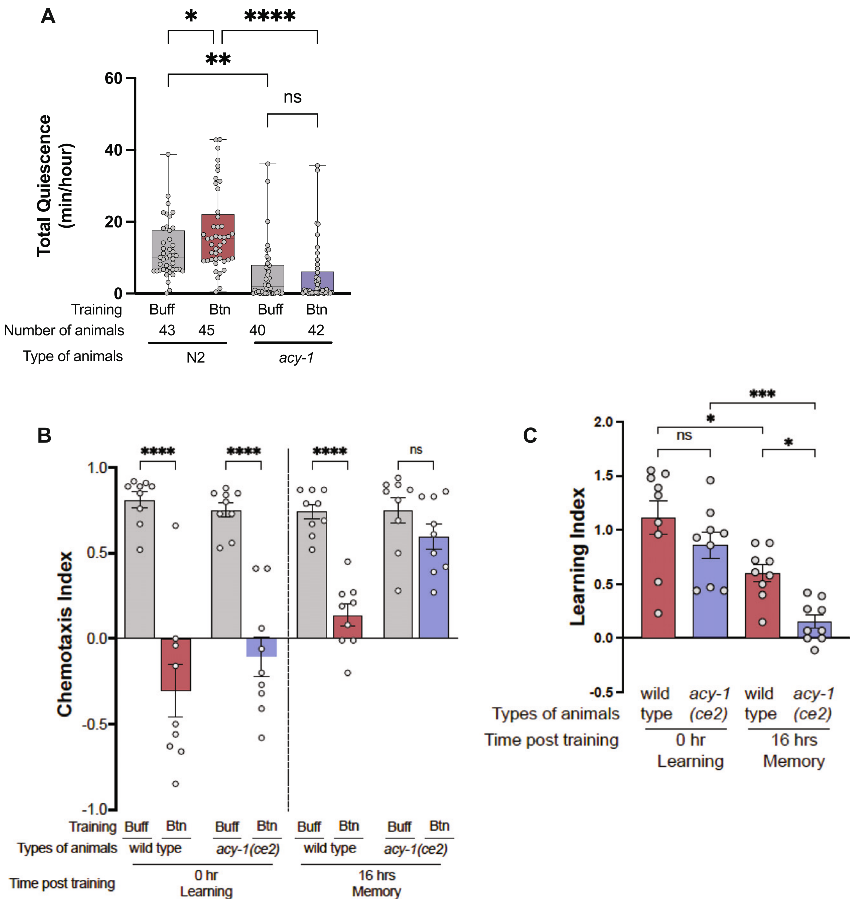

Figure S3. The sleepless *acy-1(ce2)* mutant fails to sleep after training and to keep memory, related to Figure 1

(A) *acy-1(ce2)* mutants exhibit less movement quiescence in the WorMotel than wild types after either buffer or butanone training. ****p < 0.0001, ***p < 0.001, **p < 0.01, *p < 0.05, and (ns) is p > 0.05, one-way ANOVA with Bonferroni's multiple correction. Each gray dot represents one animal, and the N below is the total number of animals examined over 5 independent days trials.

(B and C) (B) The CIs and (C) LIs of wild type and *acy-1(ce2)* are shown. ****p < 0.0001, ***p < 0.001, **p < 0.01, *p < 0.05, and (ns) is p > 0.05, two-way ANOVA with Bonferroni's multiple correction, N = 9 trials. Table S1 supports the statistics and Table S2 contains the raw and analyzed data for this figure.

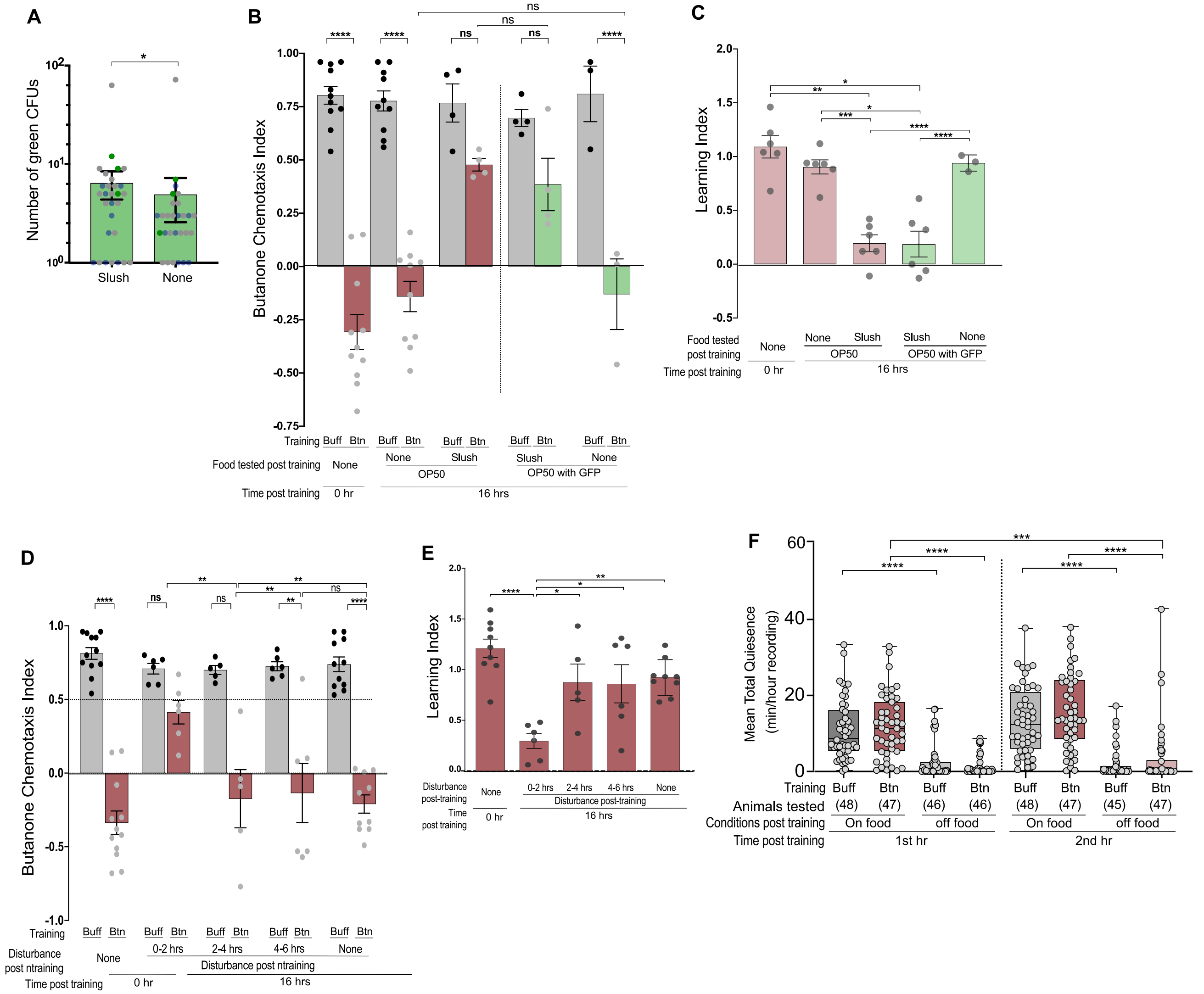

Figure S4. Placing *C. elegans* in a slurry of bacteria does not reduce feeding but does reduce sleep and removing animals from food also reduces sleep, related to Figure 3

(A) Determining if animals consume equivalent amounts of bacteria during mechanical disturbance was accomplished by counting the number of GFP-positive colony-forming units (CFUs) within the intestines of butanone-trained animals in the 2 h after training. Animals on the left (lawn, which is standard unless mentioned) were placed onto a solid lawn of GFP-expressing OP50. This was compared to the CFUs in worms placed in low viscosity GFP-expressing OP50 (slurry) that was disturbed by shaking every 15 min for 2 h. $p < 0.05$, paired t tests, n = 3 biological replicates.

(B and C) Determining how GFP-expressing OP50 2 h after training affects long-term memory was accomplished by determining the CIs and LIs after recovery with OP50 versus OP50 with GFP. There are no behavioral differences in animals that depend on the genetics of the bacteria. Instead, mechanical disturbance of worms in a slurry of either OP50 expressing or not expressing GFP after training affects memory 14 h later. ****$p < 0.0001$, ***$p < 0.001$, **$p < 0.01$, *$p < 0.05$, and (ns) is $p > 0.05$, one-way ANOVA. Each gray dot (N) represents the number of independent trials.

(D and E) Bar graphs showing the CIs and LIs of animals that were placed in the thick slurry without added mechanical disturbance for a 2-h period after training. These data show that incubating worms in the slurry in the 2 h immediately after training blocks memory at 16 h after training, but incubation in the slurry 2–4 or 4–6 h after training has no effect on 16-h memory. To obtain robust sleep and memory disruption, these slurries were also mechanically disturbed (Figures 2B and 2C). One-way ANOVA was performed, and p values are reported as ****$p < 0.0001$, ***$p < 0.001$, **$p < 0.01$, *$p < 0.05$, and (ns) is $p > 0.05$. Each gray dot (N) represents the number of independent trials.

(F) Removal from food during the first 2 h after training disrupts sleep. ****$p < 0.0001$, ***$p < 0.001$, **$p < 0.01$, *$p < 0.05$, and (ns) is $p > 0.05$, one-way ANOVA. Each gray dot (N) represents an animal. Table S1 supports the statistics and Table S2 contains the raw and analyzed data for this figure.

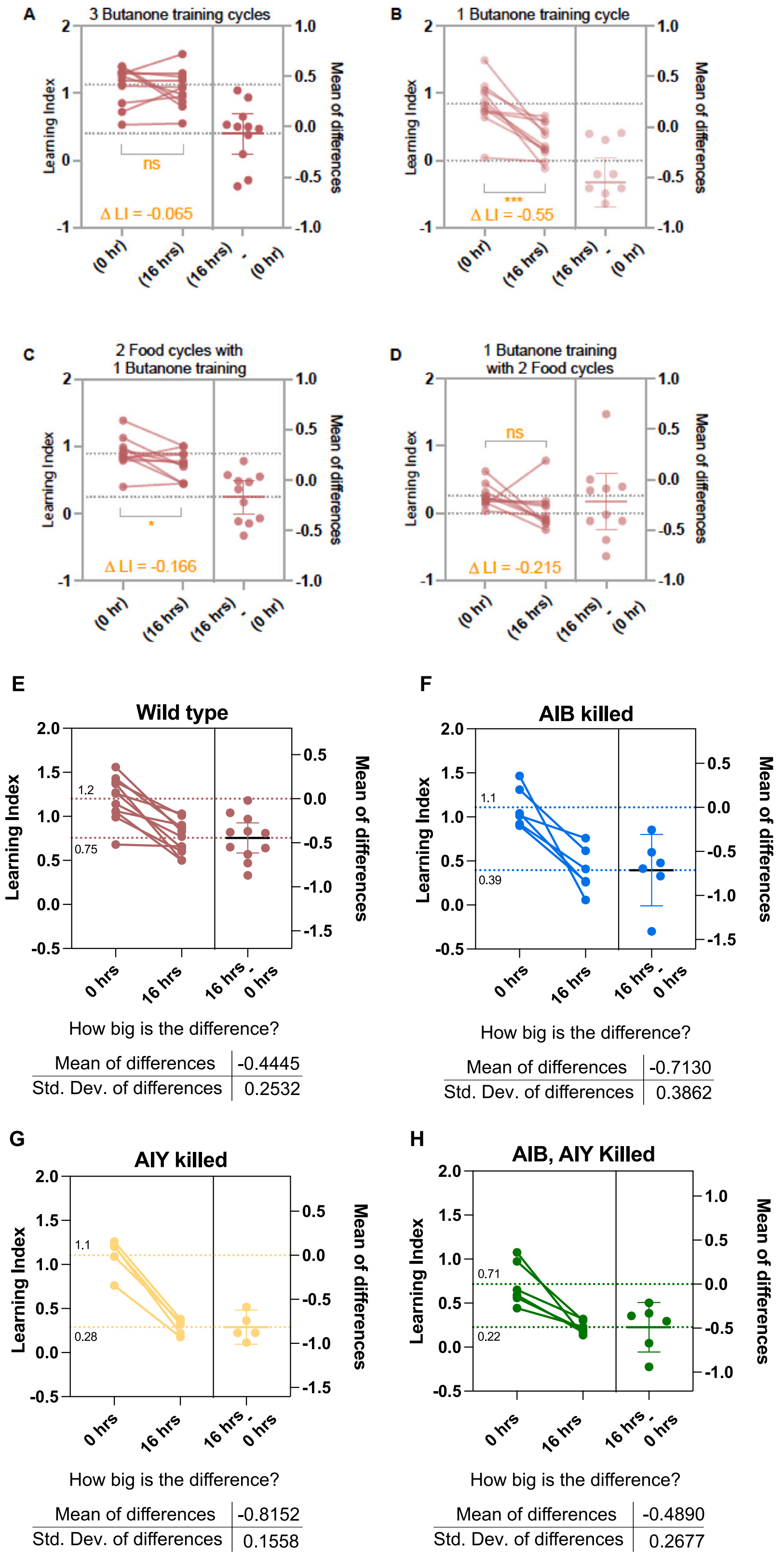

(legend on next page)

Figure S5. Learning is not appreciably lost over 16 h if animals are trained three times with butanone or once with butanone plus twice with swimming in food and more memory is lost if animals do not have the AIY interneuron, related to Figures 4 and 5

(A) One-tailed paired t tests of three butanone training cycles show that the LIs remain similar during learning (0 h) and during memory (16 h later), suggesting that LI decay is negligible. N = 10 trials.

(B) One-tailed paired t tests of one butanone training cycle show that the LIs fall significantly from learning (0 h) to memory (16 h later) suggesting that memory decays after one butanone training cycle. N = 9 trials.

(C) One-tailed paired t tests of two food cycles then one butanone training cycle show that although the LIs fall significantly from learning (0 h) to memory (16 h later), the amount of LI decay (−0.166) is much lower than just 1 butanone training cycle (−0.55). N = 10 trials.

(D) One-tailed paired t tests of one butanone training cycle plus two food cycles show that the LIs do not fall significantly from learning (0 h) to memory (16 h later) and the amount of LI decay (−0.215) is negligible. N = 10 trials. The estimation plots from the analysis are shown.

(E) The degree of depreciation in the LIs of animals with intact AIB and AIY neurons, and the average of depreciation observed in all the trials are shown. The LIs of wild-type animals decreases from an average of 1.2 to 0.75 from 0 to 16 h.

(F) The LI depreciation in animals with AIBs killed range from an average of 1.1 to 0.39, however, the range of observed LI differences are higher due to increased variability.

(G) The AIY-killed animals exhibit the biggest LI depreciation between 0 and 16 h within a range of average 1.1 LI at 0 h to 0.28 LI at 16 h with least variability.

(H) Animals with both AIB and AIY-killed exhibit the least LI loss between 0 and 16 h (average LI from 0.71 to 0.22). These data also show that they learned least; therefore, AIB- and AIY-killed animals retained least memory. All statistical tests were one-tailed paired t tests; the estimation plots from the analysis are shown. Table S1 supports the statistics and Table S2 contains the raw and analyzed data for this figure.

CellPress
OPEN ACCESS

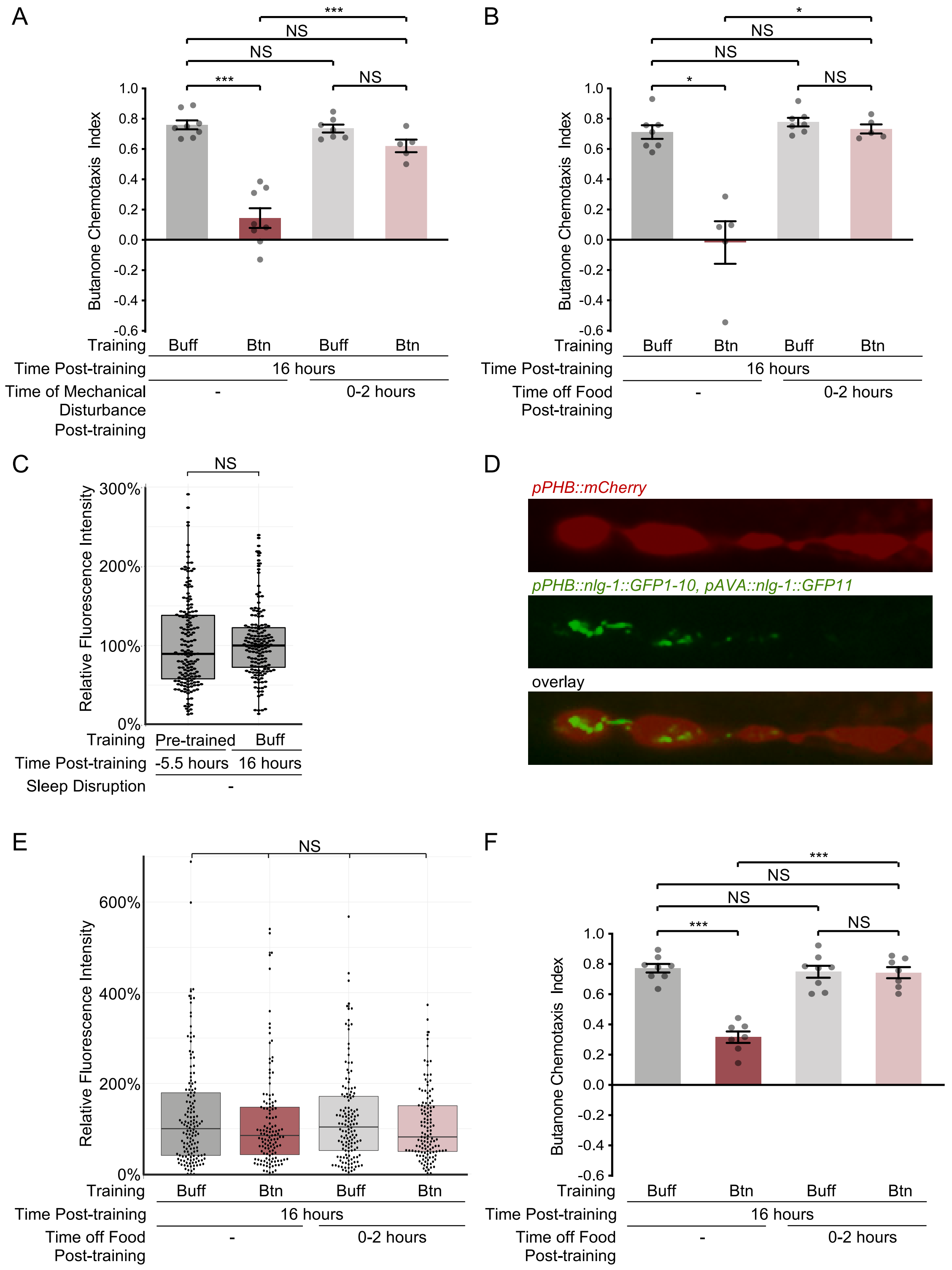

Figure S6. Animals with AWC-AIY synaptic reductions exhibit sleep-dependent memory, and AWC-AIY synaptic levels before training are similar to those in buffer-trained animals 16 h after training, and PHB-AVA synapses are not altered by training and sleep, related to Figure 6

(A and B) Chemotaxis indices of AWC-AIY NLG-1 GRASP-carrying animals trained for Figures 6D and 6F (A) and Figures 6E and 6G (B). Animals were imaged from buffer-trained (Buff) batches and butanone-trained (Btn) batches whose sleep was disrupted that sensed butanone (CI > 0.5), and from butanone-trained batches whose sleep was not disrupted that did not sense butanone well (CI < 0.5). NS $p > 0.05$, *$p < 0.05$, ***$p < 0.001$, t test. p values were adjusted for multiple comparisons using the Hochberg procedure. Error bars are SEM.

(legend continued on next page)

(C) AWC-AIY NLG-1 GRASP fluorescence intensity in animals immediately before training (5.5 h before training was complete) was similar to that observed in buffer-trained animals 16 h after training was complete. NS $p > 0.05$. p values were adjusted for multiple comparisons using the Hochberg procedure. Tables S3 and S4 support this figure.

(D) NLG-1 GRASP labeling synapses between the left and right PHB chemosensory neurons and the AVA interneurons. PHB neurons are labeled with cytosolic mCherry.

(E) Quantification of PHB-AVA NLG-1 GRASP fluorescence intensity in animals trained with buffer (Buff) or butanone (Btn) whose sleep was not disrupted (left two boxes), or whose sleep was disrupted by removal from food for 2 h immediately after training (right two boxes). There is no significant difference between the four training groups. NS $p > 0.05$, Kruskal-Wallis test.

(F) Chemotaxis indices of PHB-AVA NLG-1 GRASP-carrying animals trained for (E). Animals were imaged from buffer-trained batches and butanone-trained batches whose sleep was disrupted that sensed butanone (CI > 0.5), and from butanone-trained batches whose sleep was not disrupted that did not sense butanone well (CI < 0.5). NS $p > 0.05$, ***$p < 0.001$, t test. p values were adjusted for multiple comparisons using the Hochberg procedure. Error bars are SEM. Tables S3 and S4 support this figure.

CellPress
OPEN ACCESS

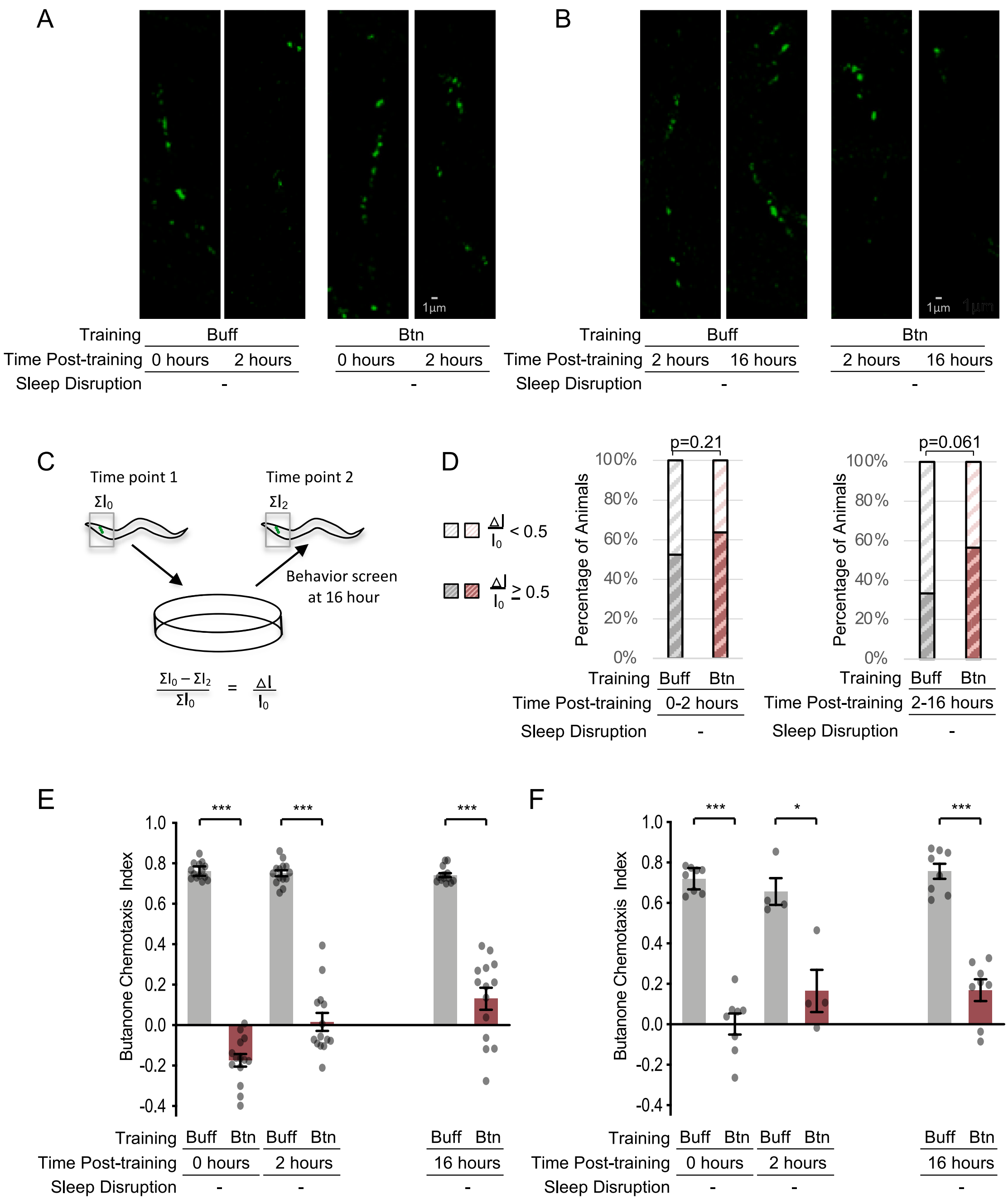

Figure S7. Single-worm synaptic imaging time course studies reveal synaptic reductions over time, and batch chemotaxis data, related to Figure 7

(A) AWC-AIY NLG-1 GRASP fluorescence in buffer-trained (Buff) animal (left) and butanone-trained (Btn) animal (right), each imaged at 0 and 2 h after training.
(B) Buffer-trained (Buff) animal (left) and butanone-trained (Btn) animal (right), each imaged at 2 and 16 h after training. (A and B) Scale bars are 1 micron.
(C) Schematic of procedure for imaging animals and quantifying the change in intensity between the time points, including single-worm behavioral screens performed 16 h after training. Individual animals imaged were from populations of buffer-trained animals that chemotaxed to butanone (CI > 0.5) or butanone-trained populations that did not chemotaxis to butanone (CI < 0.5) at 0 or 2 h. Individual buffer-trained animals that were attracted to the odor, and butanone-trained animals that were not attracted to the odor were then imaged again. For animals to pass this behavioral screen, buffer-trained worms needed to move directly toward butanone or stay on the butanone side of the plate the majority of the time, while butanone-trained worms needed to move and not chemotax toward butanone or spend the majority of time on the butanone side of the plate.
(D) Proportions of animals with large reductions (≥50%) in AWC-AIY NLG-1 GRASP fluorescence intensity between 0 and 2 h after training with buffer or butanone (left) and between 2 and 16 h (right). N > 20 for each group. p values were calculated using the z test. Similar proportions of buffer- and butanone-trained animals had a large reduction in synaptic intensity between 0 and 2 h after training, consistent with synapses being reduced after training independently of

(legend continued on next page)

whether animals are exposed to odor ($p = 0.21$, two-independent sample z test). Between 2 and 16 h, 57% of butanone-trained animals had a large synaptic reduction compared to 33% of buffer-trained animals, although these proportions were not significantly different ($p = 0.061$, two-independent sample z test).
(E and F) Batch chemotaxis indices of AWC-AIY NLG-1 GRASP-carrying animals trained for experiments in (A)–(D) and (E) and for experiments in Figure 7 (F). Animals were imaged from buffer-trained (Buff) batches and butanone-trained (Btn) batches whose sleep was disrupted that sensed butanone (CI > 0.5), and from butanone-trained batches whose sleep was not disrupted that did not sense butanone well (CI < 0.5). NS $p > 0.05$, ***$p < 0.001$, *$p < 0.05$, t test. p values were adjusted for multiple comparisons using the Hochberg procedure. Error bars are SEM. Note that for the experiments in (F), only one of each set of two training batches on each day could be tested for chemotaxis 2 h after training, given the timing constraints due to training and imaging. Tables S3 and S4 support this figure.
